# Supplementary material for: Membrane Recognition and Binding by the Phosphatidylinositol Phosphate Kinase PIP5K1A: A Multiscale Simulation Study
Source: Structure. 2019 Aug 6;27(8):1336–1346.e2. doi: 10.1016/j.str.2019.05.004 (PMC6688827; doi:10.1016/j.str.2019.05.004)
Supplement: Document S1. Figures S1–S6 [file mmc1.pdf]

**Structure, Volume *27***

## Supplemental Information

# Membrane Recognition and Binding by the Phosphatidylinositol Phosphate Kinase PIP5K1A: A Multiscale Simulation Study

**Sarah-Beth T.A. Amos, Antreas C. Kalli, Jiye Shi, and Mark S.P. Sansom**

**Supplementary Figures**

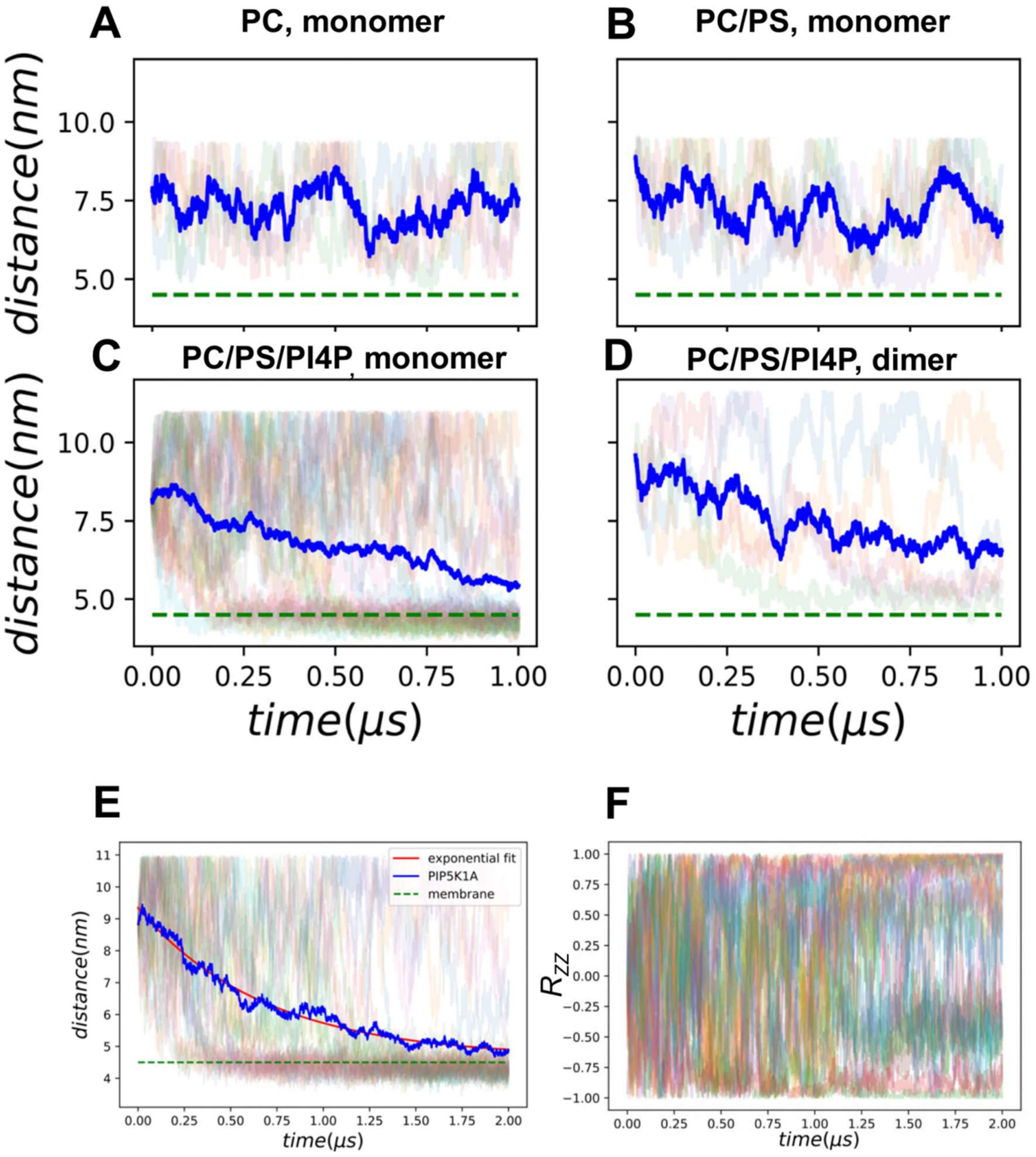


*SI Figure S1:*

Progress of the membrane association simulations shown as the distance of the kinase centre of mass from the bilayer centre vs. time, averaged over all simulations in the ensemble (see Table 1), for the: **A** PIP5K1A (monomer)/PC; **B** PIP5K1A (monomer)/PC/PS; **C** PIP5K1A (monomer)/PC/PS/PI4P; and **D** PIP5K1A (dimer)/PC/PS/PI4P simulations. The broken horizontal green line indicates the distance at which the kinase would be bound to the bilayer surface. The pale coloured lines represent the distance vs. time data for the individual simulations within each ensemble. **E** PIP5K1A (monomer)/PC/PS/PI4P, 2μs simulations. The average distance of the kinase centre of mass was fitted with a single exponential model (red curve) with a decay time of 0.7 μs. **F** $R_{zz}$ of the individual PIP5K1A monomers over time (shown via a different coloured line for each monomer), demonstrating that the protein tumbles in solution before interacting with the bilayer. (Related to Figure 2.)

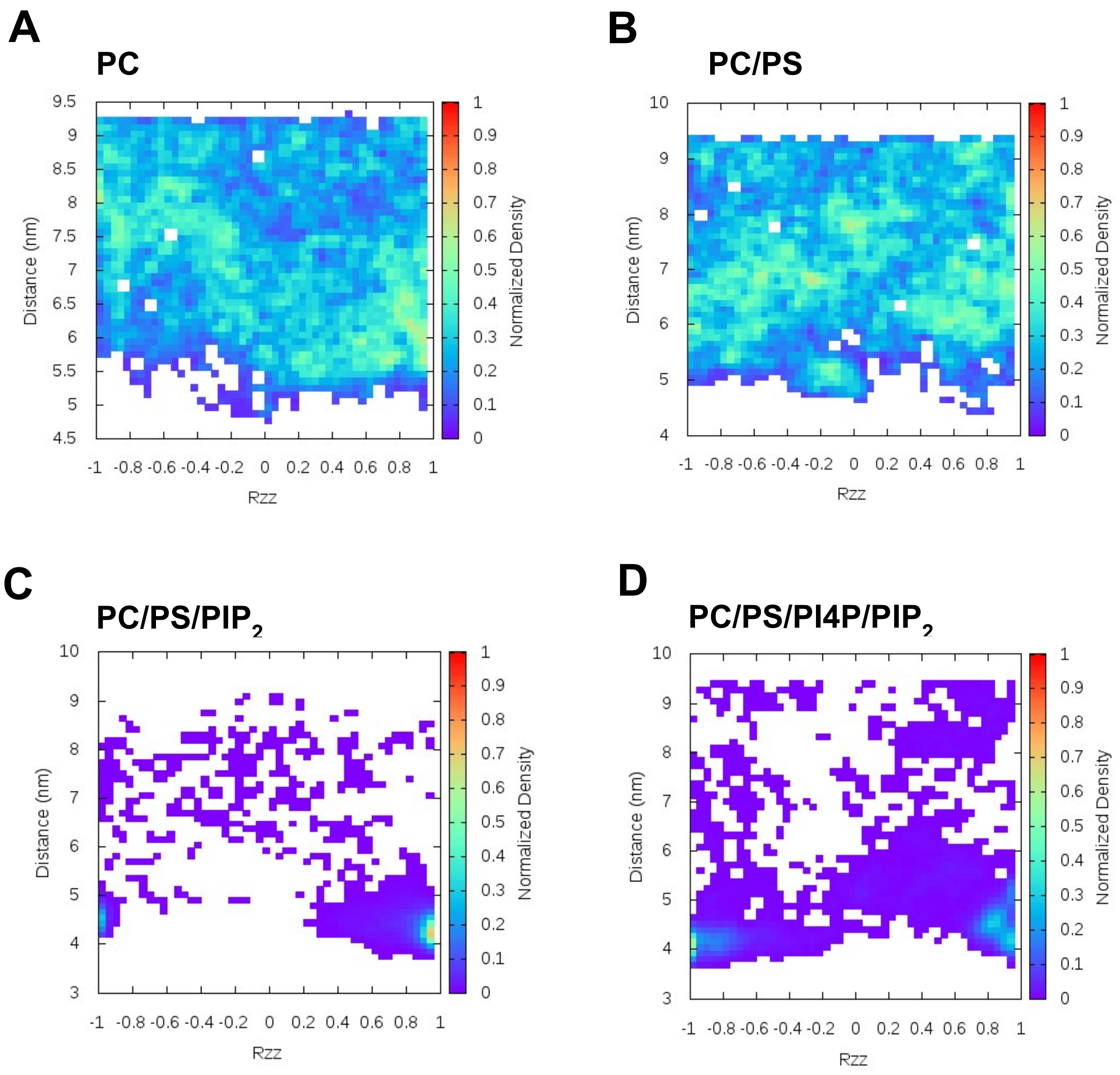


*SI Figure S2:*

**A** Orientation density map for PC control; **B** orientation density map for PC/PS; **C** orientation density map for PC/PS/PIP2; **D** orientation density map for PC/PS/PIP2/PI4P mixed. Both control simulations (i.e. **A** and **B**) show a random distribution of orientations, and therefore no specific orientations with respect to the bilayer. (Related to Figure 3.)

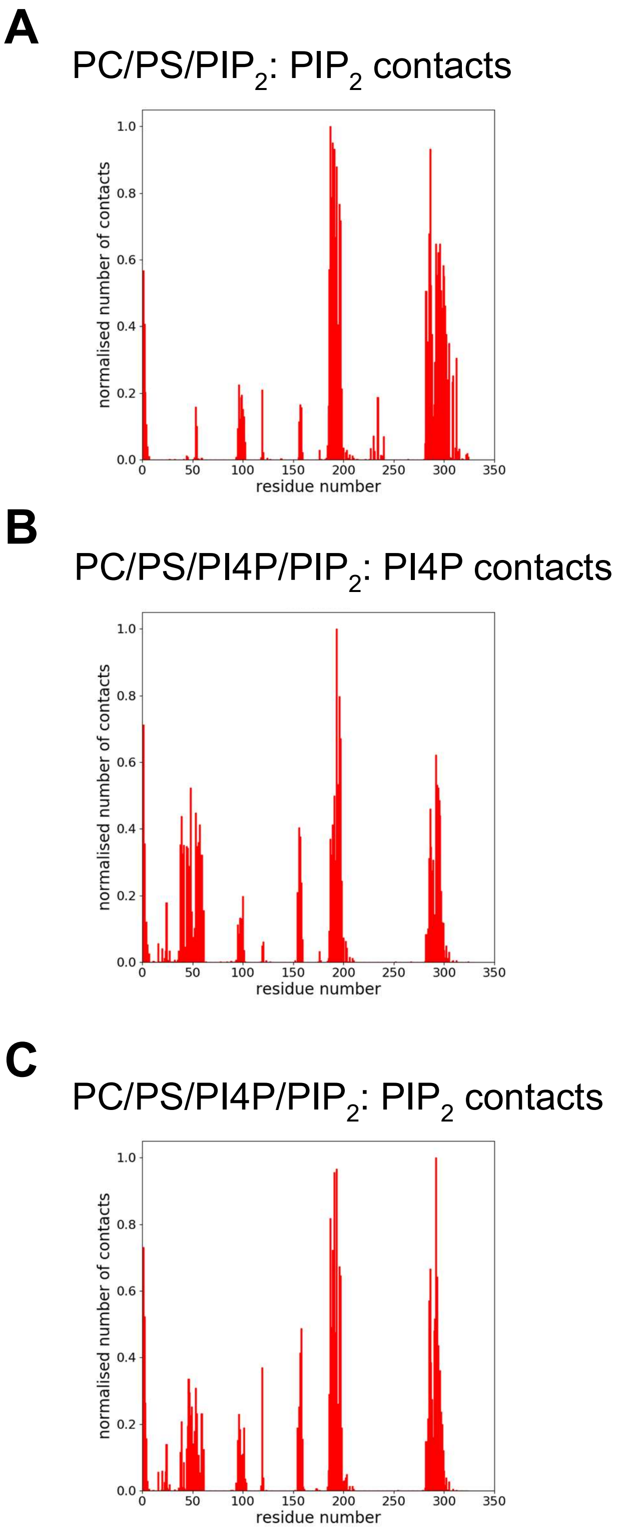


*SI Figure S3:*

**A** Normalized number of contacts between PIP5K1A residues and $PIP_2$ in a PC/PS/$PIP_2$ membrane; **B** normalized number of contacts between PIP5K1A and PI4P in a PC/PS/PI4P/$PIP_2$ membrane; **C** normalized number of contacts between PIP5K1A and $PIP_2$ in a PC/PS/PI4P/$PIP_2$ membrane. (Related to Figure 3.)

# PIP5K1A/PC/PS/PI4P-dimer-restrained-2PIP

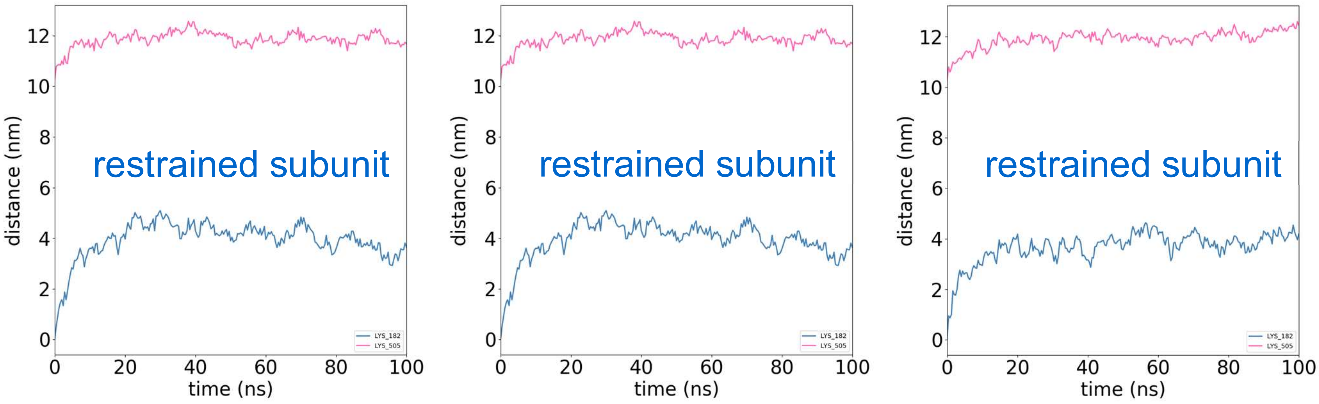


<u>*SI Figure S4:*</u>
Progression of three PIP5K1A/PC/PS/PI4P-dimer-restrained-2PIP (Table 1) simulations performed with the dimer initially positioned flat on the surface of the membrane and restraints placed between the active site lysine residue (PDB residue 328; simulation residue 182) and two PI4P molecules. When K328 residue is restrained to 2 PI4P molecules, the restrained subunit (blue line) remains bound to the membrane and the unrestrained monomer (pink line) remains unbound positioned away from the membrane in the cytoplasm. (Related to Figure 5.)

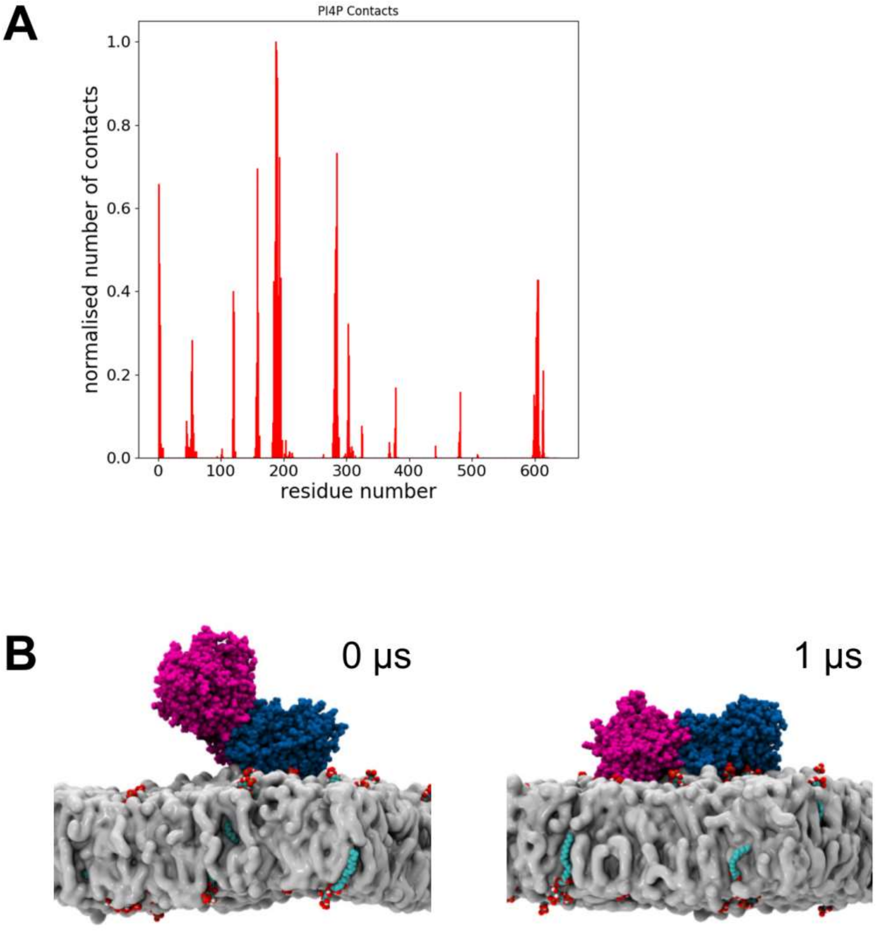


<u>*SI Figure S5:*</u>

**A** An atomistic PIP5K1A kinase dimer + bilayer system from the AT PIP5K1A/PC/PS/PI4P-dimer simulation (see Table 1) was converted to CG and run for 3 x 1 µs (simulation PIP5K1A/PC/PS/PI4P-dimer-ATconf) to determine whether PI4P could bind to both catalytic sites at the same time. **B** Snapshots of the tilted (0 µs) and flattened orientation of the dimer at the bilayer surface from the AT PIP5K1A/PC/PS/PI4P-dimer simulation. (Related to Figure 6.)

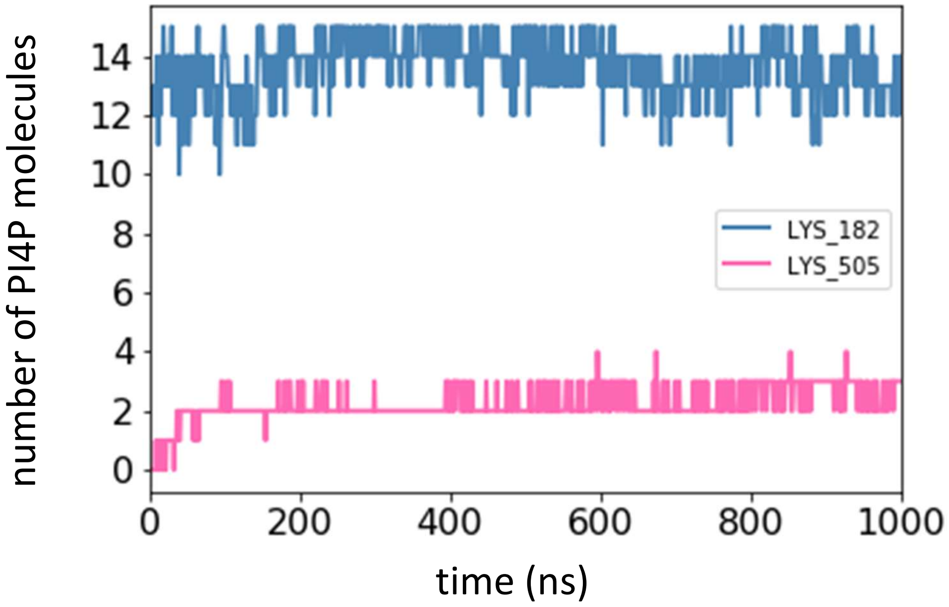


*SI Figure S6:*
Number of PI4P molecules within 3.5 nm of the active site catalytic residue over the course of the 1 atomistic simulation of the kinase dimer (PIP5K1A/PC/PS/PI4P-dimer). There are 12-14 PI4Ps clustered around the bound monomer, and around 2-4 PIPs near the active site of the other (unbound) monomer. (Related to Figure 6.)
