# Supplementary material for: Membrane Recognition and Binding by the Phosphatidylinositol Phosphate Kinase PIP5K1A: A Multiscale Simulation Study
Source: Structure. 2019 Aug 6;27(8):1336–1346.e2. doi: 10.1016/j.str.2019.05.004 (PMC6688827; doi:10.1016/j.str.2019.05.004)
Supplement: Document S2. Article plus Supplemental Information [file mmc3.pdf]

Theory

Structure

Membrane Recognition and Binding by the Phosphatidylinositol Phosphate Kinase PIP5K1A: A Multiscale Simulation Study

Graphical Abstract

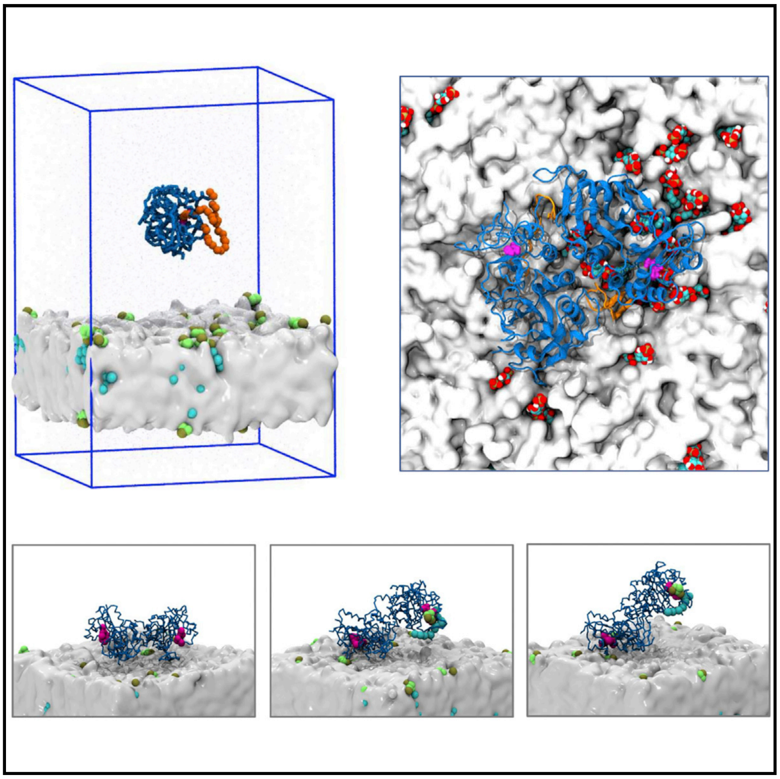

Highlights

- PIP5K1A kinase interacts with PIP-containing membranes via its activation loop
- PIP5K1A does not bind to zwitterionic or anionic membranes lacking PIP molecules
- Initial encounter of protein and bilayer is followed by reorientation and binding
- Dimeric PIP5K1A binds with membrane contacts via only one catalytic site at a time

Authors

Sarah-Beth T.A. Amos, Antreas C. Kalli, Jiye Shi, Mark S.P. Sansom

Correspondence

mark.sansom@bioch.ox.ac.uk

In Brief

Amos et al. use molecular dynamics simulations to explore interactions of PIP5K1A with PI4P-containing membranes. Kinase-bilayer encounter was followed by reorientation to enable productive binding. Dimeric kinase could not bind the membrane via both active sites simultaneously, suggesting a protein conformational change and/or bilayer distortion are needed for dual-site binding.

Amos et al., 2019, Structure *27*, 1336–1346
August 6, 2019 © 2019 The Authors. Published by Elsevier Ltd.
https://doi.org/10.1016/j.str.2019.05.004

CellPress

CellPress

Structure
Theory

Membrane Recognition and Binding by the Phosphatidylinositol Phosphate Kinase PIP5K1A: A Multiscale Simulation Study

Sarah-Beth T.A. Amos,[1] Antreas C. Kalli,[1,3] Jiye Shi,[2] and Mark S.P. Sansom[1,4,*]
[1]Department of Biochemistry, University of Oxford, South Parks Road, Oxford OX1 3QU, UK
[2]UCB Pharma, 208 Bath Road, Slough SL1 3WE, UK
[3]Present address: Leeds Institute of Medical Research at St James's, University of Leeds, Wellcome Trust Brenner Building, St James's University Hospital, Leeds LS9 7TF, UK
[4]Lead Contact
*Correspondence: mark.sansom@bioch.ox.ac.uk
https://doi.org/10.1016/j.str.2019.05.004

SUMMARY

Phosphatidylinositol phosphates (PIPs) are lipid signaling molecules that play key roles in many cellular processes. PIP5K1A kinase catalyzes phosphorylation of PI4P to form PIP_2, which in turn interacts with membrane and membrane-associated proteins. We explore the mechanism of membrane binding by the PIP5K1A kinase using a multiscale molecular dynamics approach. Coarse-grained simulations show binding of monomeric PIP5K1A to a model cell membrane containing PI4P. PIP5K1A did not bind to zwitterionic or anionic membranes lacking PIP molecules. Initial encounter of kinase and bilayer was followed by reorientation to enable productive binding to the PI4P-containing membrane. The simulations suggest that unstructured regions may be important for the preferred orientation for membrane binding. Atomistic simulations indicated that the dimeric kinase could not bind to the membrane via both active sites at the same time, suggesting a conformational change in the protein and/or bilayer distortion may be needed for dual-site binding to occur.

INTRODUCTION

The interaction of membrane lipids and proteins plays a key role in signaling within and between cells in multicellular organisms. The cell membrane comprises multiple lipid species of varying chemical composition that are subject to spatiotemporal regulation (Fernandis and Wenk, 2007) for cell signaling. A wide range of cellular functions, including signaling and trafficking, are dependent on lipid-dependent binding of peripheral membrane proteins to the plasma membrane (Lemmon, 2008). Thus, peripheral membrane proteins may carry out, e.g., lipid modifications, activation of small GTPases, or co-localization and recruitment with interacting partner proteins. The binding targets of peripheral membrane proteins are often anionic phospholipids (Stahelin et al., 2014), with considerable variety in the structure and binding mechanisms which enables control of their interactions.

Phosphatidylinositol phosphates (PIPs) are one of the major targets for directing binding of peripheral membrane proteins (Kutateladze, 2010). They constitute a small fraction of the lipid membrane but are essential for cell signaling (Di Paolo and De Camilli, 2006). For example, processes regulated by phosphatidylinositol-4,5-bisphosphate (PIP_2) include endocytosis, exocytosis, cell motility, cell adhesion, and signal transduction (McLaughlin et al., 2002). Furthermore it is becoming clear that several ion channels (Hansen, 2015) and receptors (Michailidis et al., 2011) are regulated by PIP_2. It is important that these processes are tightly regulated, and therefore it is unsurprising that lipid-metabolizing enzymes are of biomedical interest. For example, the 3-phosphatase PTEN enzyme, which catalyzes the hydrolysis of phosphatidylinositol-3,4,5-trisphosphate ($PI(3,4,5)P_3$ or PIP_3) to form PIP_2, is mutated in many cancers (Wishart and Dixon, 2002).

PIP kinases regulate the activation of various signaling pathways by catalyzing the formation of PIP_2 lipids from various lipid substrates (Heath et al., 2003). There are three known families of PIP kinases categorized as types I, II, and III. Type I and type II PIP kinases phosphorylate phosphatidylinositol-4-phosphate (PI4P) and phosphatidylinositol-5-phosphate, respectively, to form PIP_2. For example, PIP5K1A catalyzes the phosphorylation of PI4P to form PIP_2, thus playing an important role in many cell signaling processes. PIP5K1A is also implicated in e.g., prostate cancer (Drake and Huang, 2014), and the PIP5K1 family of kinases is regulated by interactions with other signaling proteins such as Talin (Di Paolo et al., 2002), Rac1 (Chao et al., 2010), ARF6 (Honda et al., 1999), and ARF1 (Jones et al., 2000).

The crystal structure of PIP5K1A (Figure 1A) was initially determined at 3.3 Å resolution (Hu et al., 2015). PIP5K1A participates in, for example, Wnt, signaling, and increased levels of the kinase contribute to increased cancer cell invasion, proliferation, and survival (Semenas et al., 2014). Comparisons with the structure of homologous kinase PIP4K2B (PDB: 1BO1) (Rao et al., 1998) and other kinases (Muftuoglu et al., 2016) have provided insights into likely mechanisms of catalysis and selectivity of the PIPKs.

The structure of the zebrafish PIP5K1A catalytic domain reveals a fold similar to that of protein kinases with a PIPK-specific

 © 2019 The Authors. Published by Elsevier Ltd.
This is an open access article under the CC BY license (http://creativecommons.org/licenses/by/4.0/).

CellPress

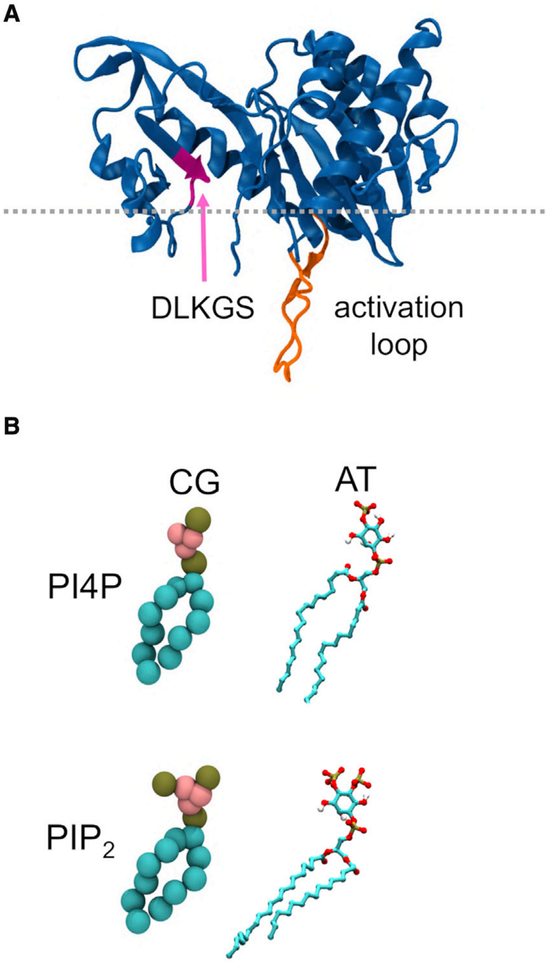

Figure 1. PIP5K1A Protein, Substrate and Product

(A) Structure of the PIP5K1A monomer (PDB: 4TZ7) with the DLKGS motif (which contributes a key lysine K238 to the active site) and the unstructured activation loop (PDB residues 378–415) labeled. The secondary structural features of the backbone atoms are in blue, the DLKGS motif in magenta, and the activation loop is in orange. The gray dotted line indicates the approximate position of the lipid membrane as revealed by subsequent simulations.

(B) Coarse-grain (CG) and atomistic (AT) representations of phosphatidylinositol the phosphates PI4P and PIP_2 (the substrate and product respectively of PIP5K1A). The tan CG particles represent phosphate groups, the pink particles represent the inositol ring, and the cyan particles represent the lipid tails. The coarse-grain representations are created using the MARTINI (Monticelli et al., 2008) force field, which uses a 4:1 mapping of non-hydrogen atoms on to coarse-grained particles. In the AT representation, tan particles represent phosphorus atoms, red represent oxygen atoms, cyan represent carbon atoms, and white represent hydrogen atoms.

subdomain that contains a "DLKGS" sequence motif. The extended DLKGS motif ($DLK_{238}GSxxxR_{244}$) is thought to form a "PIP-binding motif" (Muftuoglu et al., 2016). There is also an unstructured activation loop (also seen in PIP4K2B) that is also thought to play a role in PIP binding and selectivity. In the crystal, PIP5K1A is seen to form a side-to-side dimer, and biophysical and biochemical evidence suggest that this dimer forms in solution and is required for full catalytic activity resolution (Hu et al., 2015).

The crystal structure of PIP5K1A did not directly reveal any substrate or lipid interactions. An NMR study (Liu et al., 2016) has suggested a role of the activation loop as a molecular sensor for lipid interactions. Molecular dynamics (MD) simulations, which have been widely used to explore the interactions of both integral (Hedger and Sansom, 2016) and peripheral proteins (Kalli and Sansom, 2014) with membranes and their lipids, provide an opportunity to explore the dynamic interactions of PIPK with a PIP-containing lipid bilayer. In particular, we use multiscale MD simulations to reveal the interactions of PIP5K1A with PI4P in a bilayer, and the nature of the interactions of the PIPK dimer with the membrane surface.

RESULTS AND DISCUSSION

Binding of PIP5K1A to a Substrate-Containing Lipid Bilayer

Coarse-grained simulations were performed to explore the mode of interaction of the PIP5K1A monomer with a lipid bilayer. The protein was initially positioned away from the bilayer (Figure 2A) and multiple replicates (N = 5–25) of a 2 μs simulation performed (Table 1) in which the protein diffused within the aqueous phase before encountering and binding to the bilayer (Figures 2B and S1). We performed multiple shorter simulations rather than a single long simulation, as recent studies (e.g., Knapp et al., 2018) suggest that this may provide more effective sampling.

Simulations were performed with a bilayer containing the substrate (PI4P) as well as control simulations in which either a zwitterionic (PC) or anionic (PC/PS) bilayer without any PIP molecules present was used (see Table 1). In these simulations the protein is able to tumble in solution before encountering the membrane (Figure S1). In the PC/PS/PI4P simulations the protein encountered the bilayer typically within the first 0.5 μs before then reorienting and binding in a "productive" mode in which the DLKGS motif and the activation loop interacted with PI4P molecules at the membrane surface. In contrast, PIP5K1A did not bind to PC only or to anionic PC/PS membranes (Figure S1). Instead, in the PC/PS control simulations the kinase tumbles randomly in solution making only occasionally transient contacts with PS molecules. Recent studies of, e.g., Ras family proteins, suggest that lipid specificity is encoded by defined structures rather than just electrostatic interactions (Zhou et al., 2017), and that specific interactions with PIP_2 molecules in the membrane mediate G-Ras function (Cao et al., 2019). This supports the view that the activation loop conformation is the main determinant of lipid specificity (Kunz et al., 2000) and that this effect is captured by the coarse-grained model (Figure S1).

The PC:PS:PI4P simulations were analyzed in terms of the contacts formed to PI4P molecules by the (monomeric) kinase (Figure 3A), and of the orientation of the protein molecule relative to the bilayer to establish how the kinase interacts with the membrane. The PIP5K1A molecule made contacts with PI4P via two main regions: an arginine/lysine-rich region corresponding to the β8-α4c loop which is immediately after the DLKGS motif (PDB residues 243–253; which are simulation residues 187–197 in Figure 3) and the activation loop (PDB residues 378–415, simulation residues 276–313). A distance/orientation density map (averaged across the 25 repeat simulations; Figures 3B and S2) revealed the orientation of the kinase as a function of distance

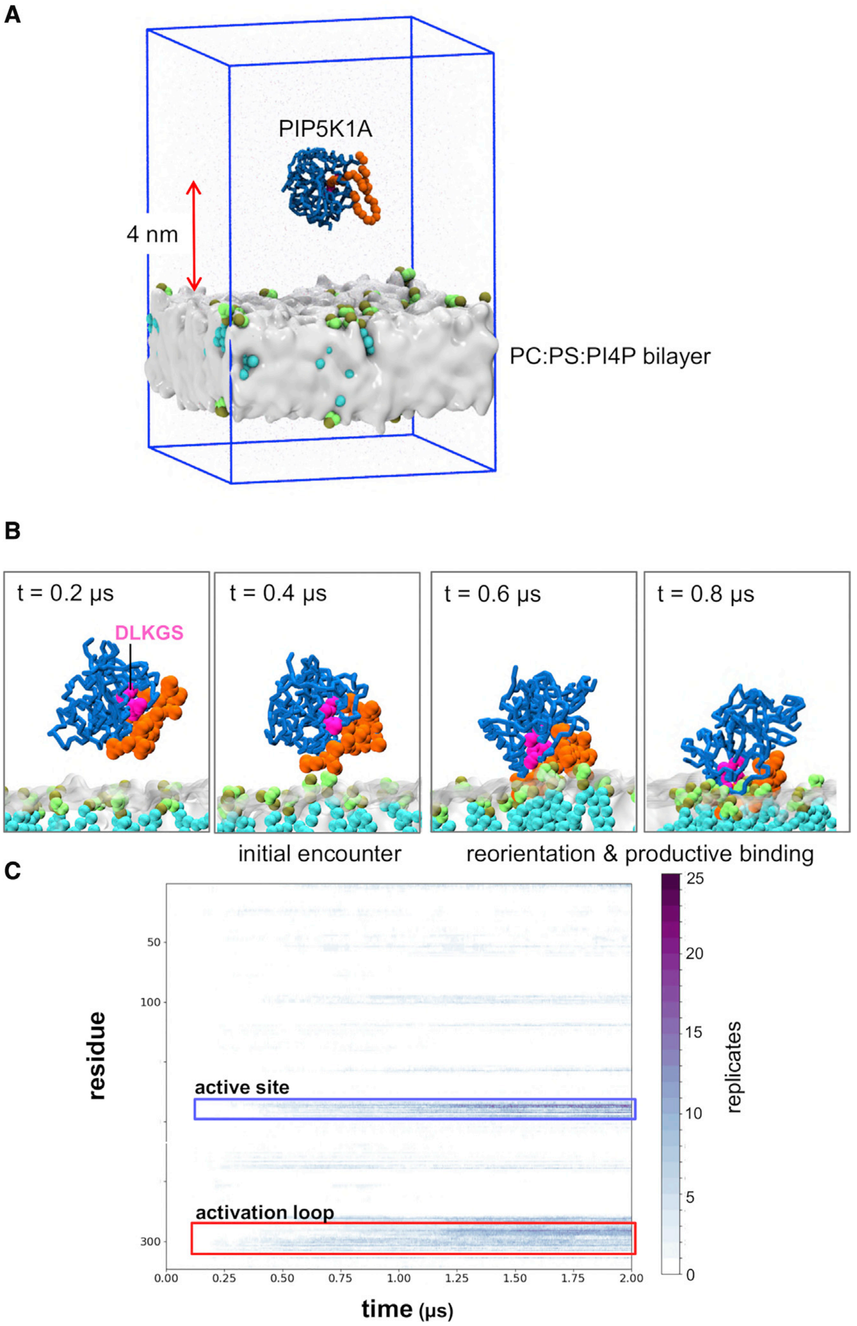

Figure 2. PIP5K1A/Membrane Encounter Simulations

(A) The initial simulation setup. The CG model of the PIP5K1A monomer was placed such that its center of mass was ~4 nm away from the surface of the lipid bilayer. The activation loop of the kinase is in orange. The blue box represents the periodic boundaries. The lipid bilayer (PC and PS) is shown in gray with PI4P molecules in green and cyan. The blue box represents the boundaries of the simulation box.

(B) An example of the progress of a CG simulation (PIP5K1A/PC/PS/PI4P; Table 1) in which the PIP5K1A molecule, initially displaced from the membrane, first encounters the bilayer. The PIP5K1A monomer first encounters the membrane, making contacts with PI4P molecules via the activation loop (orange). The PIP5K1A molecule then reorients so that both the activation loop and the DLGKS motif (pink) contact the PI4P molecules (green/cyan).

(C) Time course of protein contacts to PI4P from the PIP5K1A/PC/PS/PI4P (Table 1) simulation The activation loop (red box) makes initial contact with the membrane and is followed by reorientation and "productive binding" of DLKGS motif (which is close to the active site; blue box).

from the membrane. Two modes of interaction were seen in this map. In mode 1 the activation loop formed the main interaction with the membrane. In mode 2 (seen following reorientation of the protein on the membrane surface), contacts are also formed by the arginine/lysine-rich region noted above. This yields a model of the PIP5K1A binding in a preferred orientation for catalysis with the active site and nearby residues (including D236, K238, and R244; simulation residues 180, 182, and 188) in close contact with lipid head groups.

The activation loop is a primary determinant of substrate specificity (Kunz et al., 2000). Our simulations suggest that the activation loop (which is absent from the crystal structure and so was modeled as a flexible region) plays an important role in the binding of the kinase. It has been suggested that there is an interplay between the phosphate binding site of the substrate of the phosphorylation site (residues K238 and R244) and the activation loop (Muftuoglu et al., 2016). Our simulations indicate that K238 and R244 form major contacts to PI4P and that the activation loop retains its dynamic behavior throughout the binding process, interacting with multiple PIP molecules. Thus our results support the suggestion that the $DLK_{238}GSxxxR_{244}$ sequence (conserved among the PIPKs) corresponds to a PIP-binding motif (Muftuoglu et al., 2016).

It has also been proposed that PIP_2, the product of the phosphorylation reaction, may compete for interaction with the activation loop and therefore act as inherent feedback mechanism in the kinases (Kunz et al., 2000). In simulations of the interaction of PIP5K1A with a PIP_2-containing bilayer (Figure S3A; Table 1), we observe that it does bind to PIP_2, with a similar overall distribution of contacts. PI4P showed a slight preference for interacting with the activation loop over the active site, whereas PIP_2 showed a slight preference for the active site over the activation loop when comparing normalized numbers of contacts. The contacts made by the PIP_2 are shifted toward the N-terminus of the activation loop compared with the PI4P interactions. This suggests there may be a complex interplay of substrate and product determining the interactions and conformation of the activation loop at the surface of the bilayer, which could in principle be

Table 1. Summary of the Simulations in this Study

Simulation	Bilayer[a]	Duration (μs)	N
Coarse Grained			
PIP5K1A/PC	PC	1	5
PIP5K1A/PC/PS	PC/PS	1	5
PIP5K1A/PC/PS/PI4P	PC/PS/PI4P	2	25
PIP5K1A/PC/PS/PIP2	PC/PS/PIP_2	1	5
PIP5K1A/PC/PS/PI4P/PIP2	PC/PS/PI4P/PIP_2	1	5
PIP5K1A/PC-dimer	PC	1	5
PIP5K1A/PC/PS-dimer	PC/PS	1	5
PIP5K1A/PC/PS/PI4P-dimer	PC/PS/PI4P	1–3[b]	2 and 3[b]
PIP5K1A/PC/PS/PI4P-dimer-flat	PC/PS/PI4P	1	5
PIP5K1A/PC/PS/PI4P-dimer-NMA	PC/PS/PI4P	1	5
PIP5K1A/PC/PS/PI4P-dimer-restrained-1PIP	PC/PS/PI4P	0.1	3
PIP5K1A/PC/PS/PI4P-dimer-restrained-2PIP	PC/PS/PI4P	0.1	3
PIP5K1A/PC/PS/PI4P-dimer-restrained-3PIP	PC/PS/PI4P	0.1	3
PIP5K1A/PC/PS/PI4P-dimer-ATconf	PC/PS/PI4P	1	3
Atomistic			
PIP5K1A/PC/PS/PI4P-dimer	PC/PS/PI4P	0.10, 0.15, and 1.00	1 each

[a]Bilayer composition: 75% PC, 20% PS, and 5% PI4P (80%, 20% for PC/PS bilayers).
[b]Of the five initial simulations of 1 μs duration, three were extended to 3 μs.

explored in simulations of PIP5K1A bound to membranes with both PI4P and PIP_2 present.

Dimer Binding

Having established a detailed model of recognition of a PI4P-containing bilayer by monomeric kinase we extended our simulations to explore the mode of binding of the dimer observed in the crystal structure of PIP5K1A (Figure 4A). It is clear from biophysical and biochemical data that the side-by-side dimer forms in solution and that dimerization is required for full catalytic activity (Hu et al., 2015). However, simulations of the X-ray structure of the dimer (PIP5K1A/PC/PS/PI4P-dimer; see Table 1; Figure S4) showed that the dimer makes contacts with PI4P molecules in the membrane through either one or other of the two active sites (as defined by the DLKGS motif) on a similar timescale to that observed in comparable simulations of the monomeric kinase (Figures S1C and S1D) but not through both sites of the dimer at the same time. To test if this was due to a limitation of the simulation duration we extended the simulations to 3 μs (Table 1). However, binding of both active sites to the membrane at the same time was not observed in any of these extended simulations. Simulations were therefore performed with the dimer pre-positioned flat and just 1 nm above the bilayer (PIP5K1A/PC/PS/PI4P-dimer-flat; Table 1). In these simulations one active site quickly (within 0.1 μs) formed contacts with PI4P in the membrane, but simultaneous PI4P binding by both sites of the dimer was not observed. We reasoned this may be due to a need for some degree of flexibility in the dimer greater than that observed in the CG simulations (in which an elastic network is imposed to preserve the tertiary, but not quaternary, structure of the protein). We thus used normal mode analysis (via the elNémo server [Suhre and Sanejouand, 2004]) to estimate the low-frequency normal modes of the dimer. From the models thus generated we selected the most "flattened" conformation (Figure 4A) of the dimer and repeated our CG simulations of interactions with a bilayer using this as a starting model (simulation PIP5K1A/PC/PS/PI4P-dimer-normal modes analysis; see Table 1). In this case only two out of five simulations resulted in the dimer forming extended contacts with the membrane, and only one of these bound to the membrane for an extended period (Figure 4B). In this case, only one active site bound to a PI4P molecule in the membrane, although minimal and transient contacts made with PI4P by residues of the other monomer (Figure 4C). These contacts of the second subunit were in the activation loop, which has sufficient flexibility and length to make occasional contacts with the membrane.

To further test whether the dimer could bind to the membrane while interacting with two PI4P molecules, on bound to each monomer, we explored an initial configuration in which this was "forced" to be the case. Thus, a series of simulations (PIP5K1A/PC/PS/PI4P-dimer-restrained-1PIP, -2PIP, and -3PIP; see Table 1) were performed in which the dimer was initially positioned in a "flat" configuration on the membrane and in which distance restraints were imposed between the lysine residue of the DLKGS motif of the PI4P binding site (see above) and one, two, or three adjacent PI4P molecules in the membrane (Figures 5A and S4). In none of these simulations did the dimer adopt a stable configuration in which both subunits remained at the membrane surface. Thus, in the PIP5K1A/PC/PS/PI4P-dimer-restrained-1PIP simulations the unrestrained subunit remained at the membrane surface, and the restrained subunit moved away, while extracting the PI4P molecule out of the bilayer. In the PIP5K1A/PC/PS/PI4P-dimer-restrained-3PIP simulations, the restrained subunit remained bound to the membrane, while the unrestrained subunit dissociated from the bilayer surface (Figure 5B). In the PIP5K1A/PC/PS/PI4P-dimer-restrained-2PIP simulations, the dimer extracted both restrained PI4P molecules out of the membrane but, in one simulation, alternated between membrane binding of the two subunits (see Figure S4). Together, the various simulations may suggest that the dimer of PIP5K1A is unlikely to bind to a membrane via two PI4P molecules, one bound to each active site.

The Bound Dimer

To explore in more detail the interactions of the bound dimer, an atomistic model of the bound dimer was generated from the endpoint of the first replicate of the PIP5K1A-PC/PS/PI4P CG simulation. This was used to initiate three short atomistic simulations (0.1, 0.15, and 0.25 μs) of the bound dimer, of which the 0.25-μs simulation was extended to 1.0 μs. The simulations

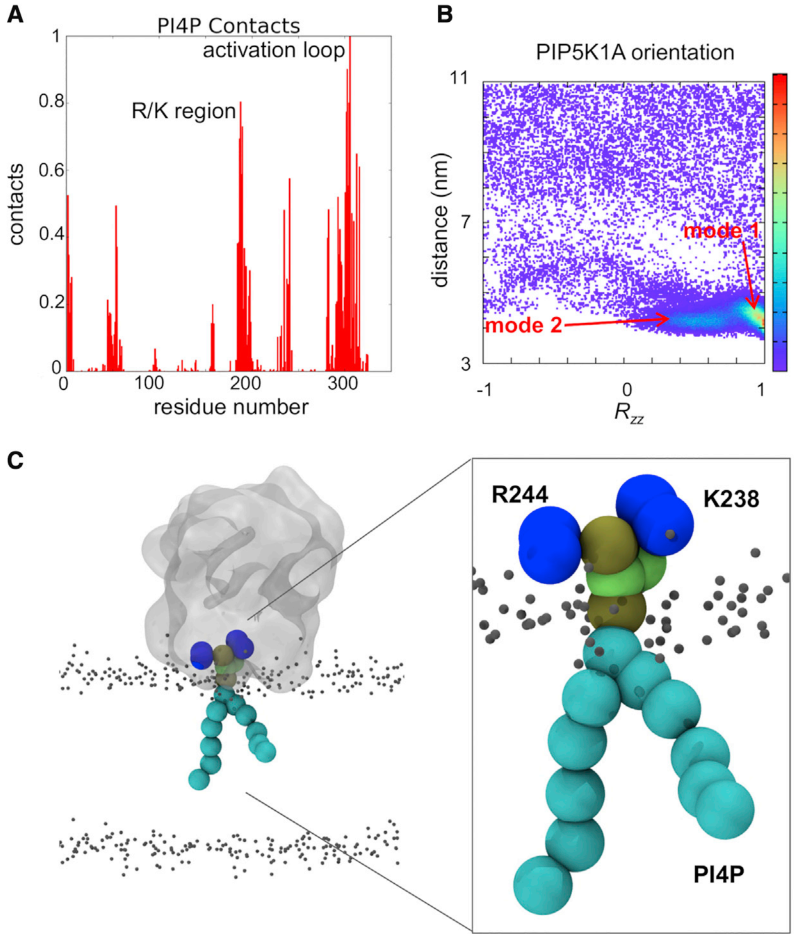

Figure 3. Membrane Contacts of PIP5K1A

(A) Contacts to PI4P for the membrane bound monomeric kinase. The normalized number of PI4P contacts averaged across all PIP5K1A/PC/PS/PI4P (Table 1) CG simulations is shown as a function of simulation residue number. (Simulation residues 1–253 correspond to PDB structure residues 57–309 and simulation residues 254–324 correspond to PDB structure residues 356–426.) The contacts are concentrated in two main regions, an arginine/lysine-rich region correspond to the β8-α4c loop, which is immediately after the DLKGS motif (PDB residues 243–253; simulation residues 187–197) and the activation loop (PDB residues 378–415; simulation residues 276–313).

(B) Density map of the distance from and orientation (given as the R_{zz} element of the orientation matrix; see main text for details) relative to the bilayer of the kinase averaged over the five PIP5K1A/PC/PS/PI4P CG simulations. Two major modes of interactions are seen: mode 1 is a little further away from the membrane, with the activation loop interacting with PIP molecules, whereas in mode 2 the kinase monomer is bound to the membrane with the DLKGS motif and the active site facing toward the surface of the bilayer.

(C) Snapshot from the end of a PIP5K1A/PC/PS/PI4P simulation showing the protein (gray; K238 and R244 in blue) bound (mode 2) to a PI4P molecule in a lipid bilayer (indicated by the phosphate particles shown as small gray spheres). The inset shows the head group of the PI4P (tan and green particles) positioned between the K238 and R244 residues (in blue).

confirmed the dimer conformation as making contacts with the membrane at one site only (Figure 6A). The pattern of PI4P contacts for the "bound" subunit was as in the CG simulations, with key contacts formed by the $DLK_{238}GSxxxR_{244}$ motif and by the activation loop (Figure 6A). Dynamic fluctuations are seen in these contacts (Figure 6C), especially for the activation loop, which shows fluctuations in its interaction with PI4Ps. On the longer (microsecond) timescale the dimer tilts toward the membrane bringing subunit B into closer contact with the membrane COM, but this does not result in substantive contacts with PI4P. In support of this we determined the number of PI4P molecules within 3.5 nm of the two active sites of the dimer (noting that the approximate radius of the monomer is 2.5 nm; see Figure S6). While there are more PI4P molecules near the bound monomer we also see that there are around 3–5 PI4Ps within 3.5 nm of the active site of the unbound monomer. This number remains fairly constant throughout the simulation. So, it seems that, while PI4P molecules are "within range" of the unbound monomer they do not interact directly with its active site. Thus, as in the CG simulations, substantive interactions with PI4P were only seen for one subunit of the dimer.

The atomistic simulations indicated a degree of flexibility in the PIP5K1A dimer: the dimer underwent hinge-bending motion at the subunit:subunit interface as demonstrated by tracking the Cα root-mean-square deviations of the individual subunits versus the dimer as a whole and via a simple angle metric (Figures 6B and S5B). This allowed both subunits to approach the membrane (Figure 6D), although binding of PI4P to the second subunit was not observed on the timescale of the atomistic simulations (Figures 6C and S6). Thus, over the time course of the simulation (Figure 6), the kinase tilts such that the unbound active site moves closer to the membrane, whereas the PI4P-bound monomer moves a little away from the membrane. This suggests that the dimer may adopt a flattened conformation on the bilayer but that this state is transient. This is seen toward the end of the 1.0-μs simulation, in which the dimer fluctuates between 100° and 120°. However, no contacts with the PI4P lipids are observed for subunit B (Figure 6C), even though several PI4P molecules were within range of the subunit (see above and Figure S6). The flattened conformation from the atomistic simulation was converted to CG and run for 3 × 1 μs to see if PIPs could bind at the catalytic site at the same time (Figure S5). The bound monomer still dominated the contact profile, although some contacts were observed in the activation loop region of the unbound monomer. However, there were still no contacts made with the catalytic lysine residue of the unbound monomer throughout the simulation. The catalytic K238 residue makes between zero and two hydrogen bonds with PI4P molecules intermittently throughout the simulation (Figure 7A). In addition, up to six hydrogen bonds are made between active site residues and

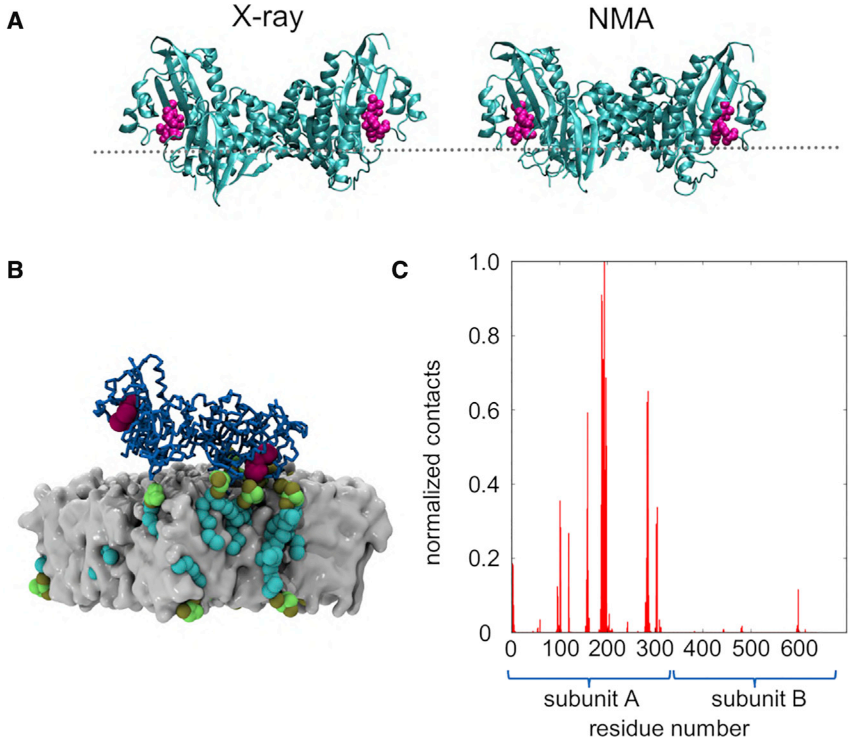

Figure 4. PIP5K1A Dimer at a Membrane Surface

(A) PIP5K1A dimer, comparing the original PDB structure (X-ray) and a model based on a low-frequency mode produced by normal modes analysis (NMA). The DLKGS motif is in pink and the approximate location of the bilayer is shown as a broken gray line.

(B) Snapshot from the PIP5K1A/PC/PS/PI4P-dimer-NMA simulation of the interaction of the dimer with a PI4P-containing membrane. It can be seen that only one subunit of the dimer forms extensive contacts to the PI4P molecules and the bilayer. While the activation loop of the unbound monomer makes some negligible contacts with PIPs in the membrane, the dimer remains bound at one site only.

(C) Normalized number of contacts between PIP5K1A NMA dimer residues and PI4P particles.

PI4P molecules. The R/K-rich region, adjacent to the catalytic site, makes multiple hydrogen bonds with PI4P molecules. In the second half of the simulation during which the dimer tilts, a concurrent loss of hydrogen bonding between PI4P molecules and the active site of subunit A is observed. In addition, the root-mean-square fluctuation (RMSF) of the Cα atoms was investigated, revealing that the regions with the lowest and least variable RMSF (0.2–0.25 nm) correspond to the regulatory C-helices. Experimental evidence suggests that membrane or substrate binding does not explain increased dimeric enzyme activity. The C-helix is an element known to be crucial for kinase regulation and it has been postulated that its tight packing in the dimer

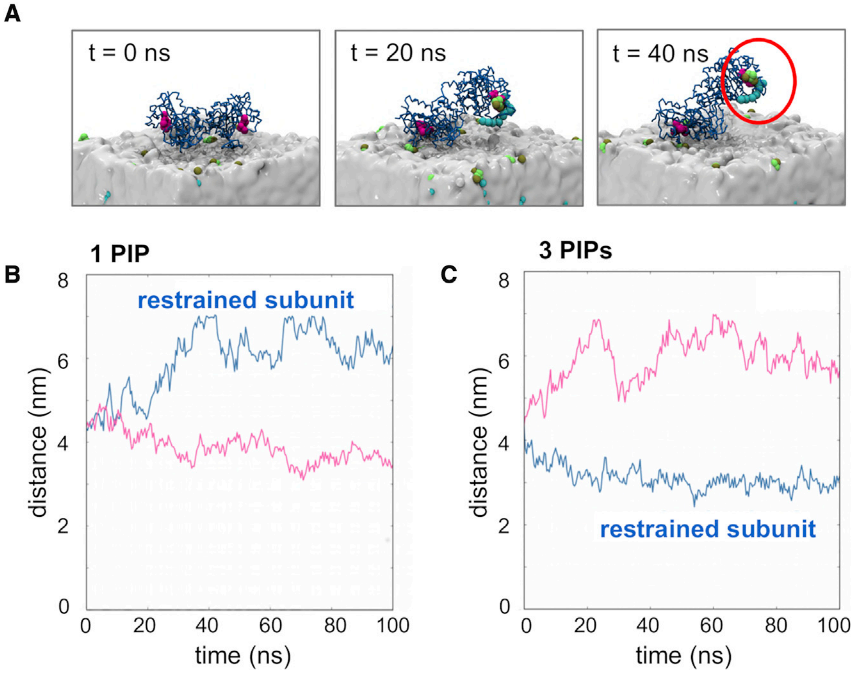

Figure 5. PIP5K1A Dimer Interactions with Pi4P at a Membrane Surface

(A) The PIP5K1A dimer binds to the PI4P membrane at either of the two active sites, but not both simultaneously, such that contacts are made at one site exclusively. The snapshots show progression of a PIP5K1A/PC/PS/PI4P-dimer-restrained-1PIP (Table 1) simulation performed with the dimer initially positioned flat on the surface of the membrane and restraints placed between the active site lysine residue (PDB residue 328; simulation residue 182) and a single PI4P molecule (the restraint was between the COMs of the residue and the PI4P head group COM). Further simulations were performed with corresponding restraints to 2 or 3 PI4P molecules (see Table 1). The restrained lipid (in cyan and green) is extracted from the membrane.

(B and C) When restrained to a single PI4P molecule (B) (1 PIP) the dimer immediately (<0.1 μs) shows a preference for one of the sites and pulls the restrained PI4P molecule (blue line) away from the membrane. The distance reported is the distance of the catalytic lysine residue (either simulation residue 182 or 505) from the membrane COM. When K328 is restrained to 3 PIPs (C), the restrained monomer (blue line) remains bound to the membrane, whereas the unrestrained monomer is detached from the membrane, as shown by the distance of the lysine residue from the membrane COM). When the lysine residue is restrained to 2 PI4P molecules, the dimer remains bound to the membrane and the unrestrained monomer remains unbound in the cytoplasm.

See Figure S3.

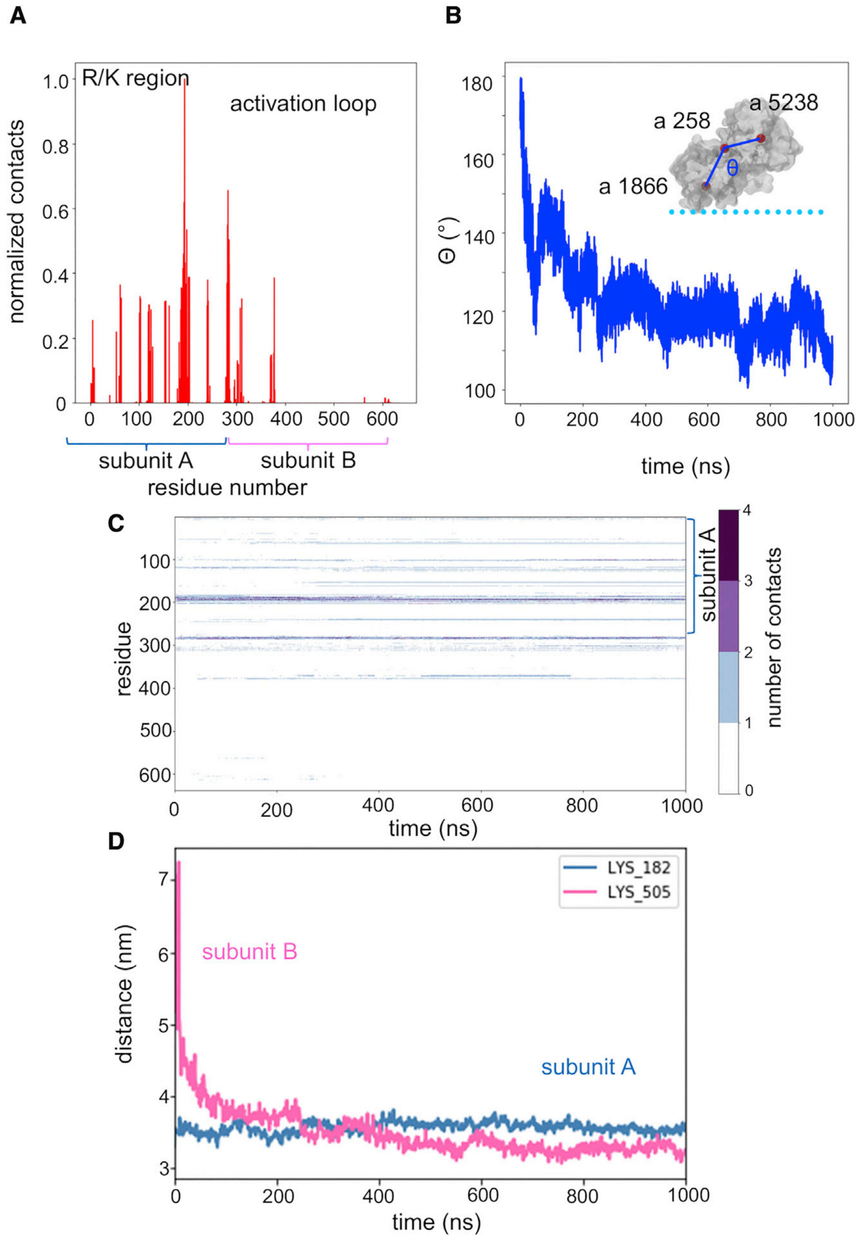

Figure 6. Atomistic Simulations of the PIP5K1A Dimer Bound to the PI4P Containing Membrane

(A) Normalized number of contacts between PIP5K1A residues and PI4P molecules in the membrane. The distribution of contacts remains similar. It is notable that, in this replicate, the middle of the activation loop has folded up out of the membrane with the end residues making numerous contacts with PI4P.

(B) Angle metric (θ) describing the orientation of the two subunits in the simulated dimer with respect to each other over time. The inset shows a schematic of the definition of θ (defined by atoms 1,866, 358, 5,238 in residues K182, V29, K505). The angle decreases from 180° at the start of simulation (corresponding closely to the crystallographic dimer) to ~140° where it remains for most of the simulation with transient periods around 110°.

(C) Contacts as defined by a 0.35 nm cutoff between the residue COM and the PI4P head group COM show PIPs collecting near the active site (DLKGS, simulation residues 180–184), while PIP contacts with the activation loop (simulation residues 280–300) remain fairly constant. Note that, with the exception of a small number of contacts made with PI4P by the activation loop in subunit B at the start of the simulation, there are no major contacts made by subunit B with PI4P.

(D) In contrast to the CG dimer simulation, the dimer was observed to bend at the dimerization interface site to bring the second monomer 4 nm closer to the membrane as shown by subunit B moving from a distance of 7 nm away from the membrane COM to 3 nm in the second half of the simulation. Taken with the angle metric (B), this reveals that the dimer tilts into the membrane bringing subunit B in closer contact with the membrane COM, but this does not result in contacts with PI4P.

See Figure S6 and Table 1.

interface results in conformational changes in the monomer that result in increased kinase activity (Hu et al., 2015) (Figure 7B). A lower level of structural variation in these regions of an otherwise dynamic protein would be consistent with this region of the structure being important for regulation and it is striking that this is revealed in the simulations.

Dimerization has been proposed (Hu et al., 2015) to increase the binding affinity of the kinase to the membrane by doubling the basic surface area that is able to make contacts with the acidic lipid head groups in the membrane, in addition to creating a flat surface that is complementary to the plane of the membrane. It has been shown in liposome flotation assays that the monomeric and dimeric binding is similar, and therefore that dimerization does not cause higher catalytic activity through membrane binding at both sites, but rather increases activity by keeping the kinase in a productive orientation. From the simulation results it would appear that the dimeric PIP5K1A kinase does not bind lipid substrates concurrently at both sites, which is consistent with an explanation that is based around key structural features keeping the kinase in a productive orientation as indicated by the RMSF profiles of the atomistic dimer simulations.

Conclusions

Our simulations provide a dynamic model of the productive orientation of PIP5K1A at the surface of a PI4P-containing lipid bilayer. One monomer (A in Figure 8) is bound to a substrate molecule with further PI4P molecules clustered about the "footprint" of the dimer on the membrane. It is clear from our analysis of the interactions of PIP5K1A with the bilayer that: (1) the DLK$_{238}$GSxxxR$_{244}$ sequence (the PIP-binding motif of Muftuoglu et al., 2016) and the activation loop form the main contacts of the enzyme with PI4P molecules in the membrane; (2) the K$_{238}$ and R$_{244}$ residues form the binding site for the 4′-phosphate of

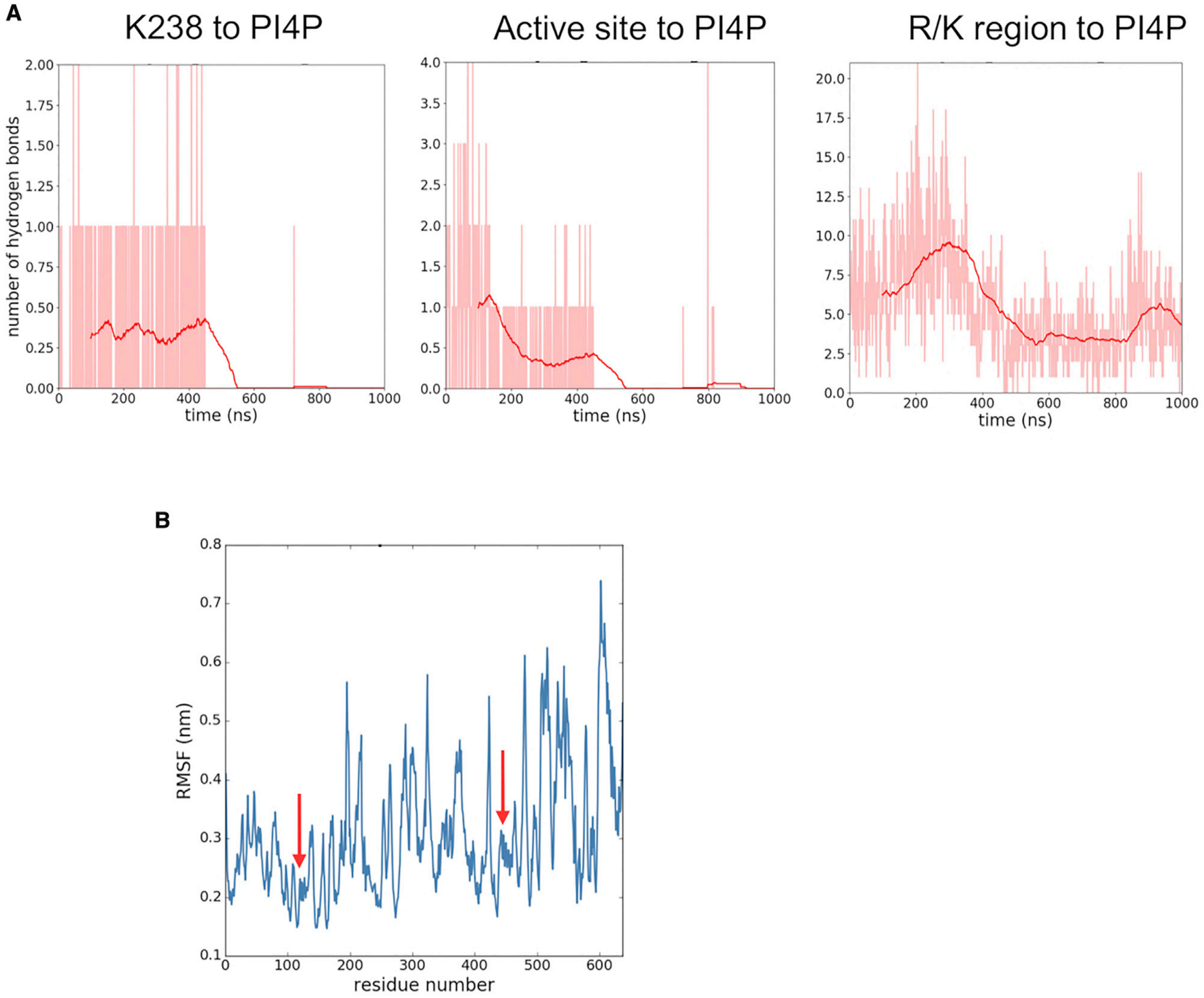

Figure 7. Atomistic Simulation of Membrane-Bound PIP5K1A Dimer

(A) Hydrogen bonding interactions to PI4P for an atomistic simulation of the PIP5K1A dimer bound to a PC/PS/PI4P membrane (see Table 1). Hydrogen bonding (shown as instantaneous numbers in pink and as moving averages in red) for a PIP5K1A dimer bound to a PC/PS/PI4P membrane simulated over 1 μs. The K238 residue makes between 0 and 2 hydrogen bonds with PIPs in the membrane. Other residues in the active site also contribute to hydrogen bonding. Residues in the R/K-rich region are involved in an extensive hydrogen bonding network which shows small fluctuations over time but remains broadly between 5 and 10 hydrogen bonds over the course of the simulation while the dimer is bending.

(B) RMSF as a function of residues number for a PIP5K1A dimer bound to a PC/PS/PI4P membrane. The arrows show the regions corresponding to the C helix that show reduced mobility.

PI4P; and (3) although the dimeric protein can sit more or less flat on the bilayer surface, it can only interact tightly with one PI4P molecule at a time.

While we may speculate that substrate binding occurs one site at a time, our studies that show no simultaneous binding across any simulations. It is quite possible that on longer (i.e., >1 μs timescales) one monomer could detach from the membrane and the dimer (stochastically) switch to binding via the other monomer. This could in turn result in a piston-like mechanism whereby dissociation of one subunit allows substrate to diffuse in/product to diffuse out. Such a mechanism might be advantageous if nano clusters of PI4P become locally exhausted due to enzyme activity. Furthermore, while the dimeric protein does not appear to bind planar membranes at both sites simultaneously, binding of the dimer to curved membranes might enable both active sites to interact. This will be investigated further in future simulations of large membranes with more complex lipid compositions (and multiple copies of bound dimer) which can undergo dynamic fluctuations in curvature (Fowler et al., 2016; Koldsø et al., 2014).

Questions remain around the dynamic behavior of the activation loop. An NMR study (Liu et al., 2016) of an isolated peptide commensurate with the PIP5K1A activation loop indicated that the unstructured region folded into an amphipathic helix in DHPC micelles and provided evidence that the activation loop functions as a membrane sensor. However, this study did not address the structure of the activation loop in a more complex

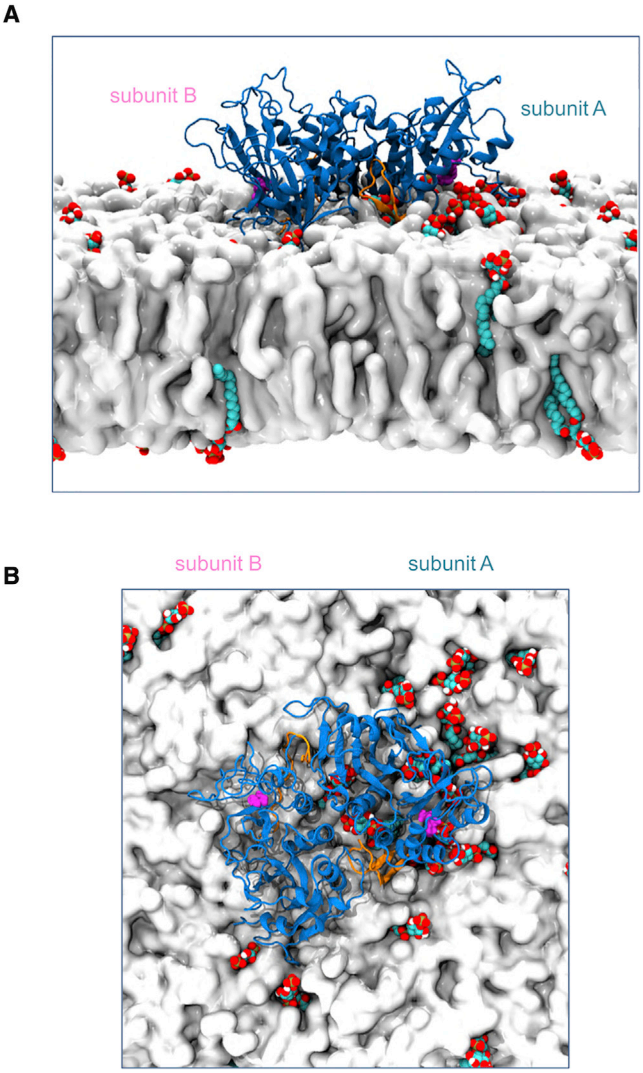

Figure 8. Final Configuration of the Membrane-Bound Dimer

(A) Snapshot of the dimer bound to the membrane after 1 μs of atomistic simulation. The kinase is shown in blue with the catalytic motif highlighted in magenta and the activation loops highlighted in orange. PI4P lipids are shown in red, white, and cyan and PC/PS lipids are shown in white for clarity. A number of PI4P molecules can be seen clustering around the bound monomer's catalytic site, whereas the unbound monomer remains close to the membrane but not bound to any PI4P molecules.

(B) Snapshot of the bound dimer viewed from above. Multiple PIPs are seen clustered around the bound subunit A with no PI4P molecules in contact with subunit B.

membrane lipid composition, and/or within the large protein structure bound to a membrane.

Limitations of the current study may include the convergence of the dimer simulations, and whether or not enough time is allowed for the flattening of the dimer onto the bilayer. However, given that, in the simulations, the dimer flattens and then relaxes, we consider that the limit of this flattening has been reached. In the 1-μs atomistic simulation this is observed twice, with no concurrent binding of PI4P to the catalytic site of the second subunit observed. Taken together with the CG simulations, the results of our multiscale simulation approach suggest that the dimeric protein cannot bind PI4P molecules at both active sites, while the lipids are in a planar membrane and the protein adopts the crystallographic dimer conformation. For the dimer to bind PI4P at both sites at the same time, our results suggest that this would require either significant distortion of the bilayer and/or a significant change in the dimer interface from the crystal structure. This is relevant to our more general understanding of the interactions of complex peripheral proteins with membranes, revealing the need to take account dynamic of protein/membrane interactions.

Finally, while the MARTINI2 model has been suggested to be too "sticky" to model protein-protein interactions (see Javanainen et al., 2017; Stark et al., 2013, but also see Domański et al., 2017), there is a growing body of evidence that suggests simulated protein-*lipid* interactions reproduce experimental results reasonably well, both within (Arnarez et al., 2013a, 2013b; Hedger et al., 2016; Schmidt et al., 2013) and at the surface (Naughton et al., 2016, 2018) of membranes. There is scope for future studies of atomistic free energy calculations to compare the relative strengths of PI4P and PIP_2 binding and also the contribution of residues in the activation loop to specificity. As noted above, it will also be of interest simulate larger coarse-grained membranes that are more complex (i.e., *in vivo* mimetic) in their lipid composition in order to investigate the influence of cholesterol and other lipids on dynamic bilayer fluctuations and PIP5K1A interactions.

Overall, we have shown that PIP5K1A can bind to the cell membrane as either a monomer or a dimer. PIP5K1A binds in a productive orientation for catalysis, such that the activation loop binds to PIP molecules and aids in the orientation of the kinase. The lipid head groups are therefore positioned close to the catalytic site for phosphorylation. Atomistic simulations reveal details of the catalytic conformation of the dimer for catalysis and the role of individual residues. In particular, we have shown that the activation loop of the PIP5K1A kinase leads the recognition of and binding to the membrane, and that a simple model of simultaneous binding of the dimer catalytic sites to the membrane is not adequate to explain how the dimeric kinase increases rates of catalysis. In the future, it could be of interest to extend these studies to bilayers that model the asymmetric lipid composition between the two leaflets of the bilayer (Ingolfsson et al., 2014), and at different concentrations corresponding to pre- and post-stimulus PIP levels in order to capture some of the complexity of signaling events.

STAR★METHODS

Detailed methods are provided in the online version of this paper and include the following:

- KEY RESOURCES TABLE
- CONTACT FOR REAGENT AND RESOURCE SHARING
- METHOD DETAILS
  - Modelling
  - Membrane Binding Simulations
  - Analysis
- QUANTIFICATION AND STATISTICAL ANALYSIS
- DATA AND SOFTWARE AVAILABILITY

CellPress

SUPPLEMENTAL INFORMATION

Supplemental Information can be found online at https://doi.org/10.1016/j.str.2019.05.004.

ACKNOWLEDGMENTS

S.-T.A.A. acknowledges the BBSRC, UCB, and Wadham College (Oxford) for funding. Research in M.S.P.S.'s group is supported by grants from Wellcome and the BBSRC. This project made use of time on ARCHER granted by the UK High-End Computing Consortium for Biomolecular Simulation, HECBioSim (www.hecbiosim.ac.uk), supported by EPSRC (grant no. EP/R029407/1). Our thanks to Matthieu Chavent for useful discussions on simulation analysis approaches.

AUTHOR CONTRIBUTIONS

S.-T.A.A. set up, performed, and analyzed the simulations. A.C.K. developed initial simulation protocols. S.-T.A.A. produced visualizations. S.-T.A.A., A.C.K., J.S., and M.S.P.S. designed the project.

DECLARATION OF INTERESTS

The authors declare no competing interests.

Received: November 28, 2018
Revised: March 7, 2019
Accepted: May 14, 2019
Published: June 13, 2019

REFERENCES

Arnarez, C., Marrink, S.J., and Periole, X. (2013a). Identification of cardiolipin binding sites on cytochrome c oxidase at the entrance of proton channels. Sci. Rep. *3*, 1263.

Arnarez, C., Mazat, J.-P., Elezgaray, J., Marrink, S.-J., and Periole, X. (2013b). Evidence for cardiolipin binding sites on the membrane-exposed surface of the cytochrome bc1. J. Amer. Chem. Soc. *135*, 3112–3120.

Berendsen, H.J.C., Postma, J.P.M., van Gunsteren, W.F., DiNola, A., and Haak, J.R. (1984). Molecular dynamics with coupling to an external bath. J. Chem. Phys. *81*, 3684–3690.

Cao, S., Chung, S., Kim, S., Li, Z., Manor, D., and Buck, M. (2019). K-Ras G-domain binding with signaling lipid phosphatidyl inositol (4,5) phosphate (PIP2): membrane association, protein orientation and function. J. Biol. Chem. *294*, 7068–7084.

Chao, W.T., Daquinag, A.C., Ashcroft, F., and Kunz, J. (2010). Type I PIPK-alpha regulates directed cell migration by modulating Rac1 plasma membrane targeting and activation. J. Cell Biol. *190*, 247–262.

Darden, T., York, D., and Pedersen, L. (1993). Particle mesh Ewald - an N.log(N) method for Ewald sums in large systems. J. Chem. Phys. *98*, 10089–10092.

de Jong, D.H., Singh, G., Bennett, W.F.D., Arnarez, C., Wassenaar, T.A., Schäfer, L.V., Periole, X., Tieleman, D.P., and Marrink, S.J. (2013). Improved parameters for the Martini coarse-grained protein force field. J. Chem. Theor. Comput. *9*, 687–697.

Di Paolo, G., and De Camilli, P. (2006). Phosphoinositides in cell regulation and membrane dynamics. Nature *443*, 651–657.

Di Paolo, G., Pellegrini, L., Letinic, K., Cestra, G., Zoncu, R., Voronov, S., Chang, S.H., Guo, J., Wenk, M.R., and De Camilli, P. (2002). Recruitment and regulation of phosphatidylinositol phosphate kinase type 1 gamma by the FERM domain of talin. Nature *420*, 85–89.

Domański, J., Hedger, G., Best, R., Stansfeld, P.J., and Sansom, M.S.P. (2017). Convergence and sampling in determining free energy landscapes for membrane protein association. J. Phys. Chem. B *121*, 3364–3375.

Drake, J.M., and Huang, J.T. (2014). PIP5K1 alpha inhibition as a therapeutic strategy for prostate cancer. Proc. Natl. Acad. Sci. U S A *111*, 12578–12579.

Fernandis, A.Z., and Wenk, M.R. (2007). Membrane lipids as signaling molecules. Curr. Opin. Lipidol. *18*, 121–128.

Fiser, A., and Sali, A. (2003). Modeller: generation and refinement of homology-based protein structure models. Methods Enzymol. *374*, 461–491.

Fowler, P.F., Chavent, M., Duncan, A., Helie, J., Koldsø, H., and Sansom, M.S.P. (2016). Membrane stiffness is modified by integral membrane proteins. Soft Matter *12*, 7792–7803.

Hansen, S.B. (2015). Lipid agonism: the PIP2 paradigm of ligand-gated ion channels. Biochim. Biophys. Acta *1851*, 620–628.

Heath, C.M., Stahl, P.D., and Barbieri, M.A. (2003). Lipid kinases play crucial and multiple roles in membrane trafficking and signaling. Histol. Histopathol. *18*, 989–998.

Hedger, G., Rouse, S.L., Domański, J., Chavent, H., Koldsø, H., and Sansom, M.S.P. (2016). Lipid loving ANTs: molecular simulations of cardiolipin interactions and the organization of the adenine nucleotide translocase in model mitochondrial membranes. Biochemistry *55*, 6238–6249.

Hedger, G., and Sansom, M.S.P. (2016). Lipid interaction sites on channels, transporters and receptors: recent insights from molecular dynamics simulations. Biochim. Biophys. Acta *1858*, 2390–2400.

Hess, B., Bekker, H., Berendsen, H.J.C., and Fraaije, J.G.E.M. (1997). LINCS: a linear constraint solver for molecular simulations. J. Comput. Chem. *18*, 1463–1472.

Hess, B., Kutzner, C., van der Spoel, D., and Lindahl, E. (2008). GROMACS 4: algorithms for highly efficient, load-balanced, and scalable molecular simulation. J. Chem. Theory Comput. *4*, 435–447.

Honda, A., Nogami, M., Yokozeki, T., Yamazaki, M., Nakamura, H., Watanabe, H., Kawamoto, K., Nakayama, K., Morris, A.J., Frohman, M.A., and Kanaho, Y. (1999). Phosphatidylinositol 4-phosphate 5-kinase alpha is a downstream effector of the small G protein ARF6 in membrane ruffle formation. Cell *99*, 521–532.

Hu, J., Yuan, Q.Y., Kang, X., Qin, Y.B., Li, L., Ha, Y., and Wu, D.Q. (2015). Resolution of structure of PIP5K1A reveals molecular mechanism for its regulation by dimerization and dishevelled. Nat. Commun. *6*, 8205.

Humphrey, W., Dalke, A., and Schulten, K. (1996). VMD - visual molecular dynamics. J. Mol. Graph. *14*, 33–38.

Hunter, J.D. (2007). Matplotlib: a 2D graphics environment. Comput. Sci. Eng. *9*, 90–95.

Ingolfsson, H.I., Melo, M.N., van Eerden, F.J., Arnarez, C., Lopez, C.A., Wassenaar, T.A., Periole, X., de Vries, A.H., Tieleman, D.P., and Marrink, S.J. (2014). Lipid organization of the plasma membrane. J. Am. Chem. Soc. *136*, 14554–14559.

Javanainen, M., Martinez-Seara, H., and Vattulainen, I. (2017). Excessive aggregation of membrane proteins in the Martini model. PLoS One *12*, e0187936.

Jones, D.H., Morris, J.B., Morgen, C.P., Kondo, H., Irvine, R.F., and Cockcroft, S. (2000). Type I phosphatidylinositol 4-phosphate 5-kinase directly interacts with ADP-ribosylation factor 1 and is responsible for phosphatidylinositol 4,5-bisphosphate synthesis in the Golgi compartment. J. Biol. Chem. *275*, 13962–13966.

Kalli, A.C., and Sansom, M.S.P. (2014). Interactions of peripheral proteins with model membranes as viewed by molecular dynamics simulations. Biochem. Soc. Trans. *42*, 1418–1424.

Knapp, B., Ospina, L., and Deane, C.M. (2018). Avoiding false positive conclusions in molecular simulation: the importance of replicas. J. Chem. Theory Comput. *14*, 6127–6138.

Koldsø, H., Shorthouse, D., Hélie, J., and Sansom, M.S.P. (2014). Lipid clustering correlates with membrane curvature as revealed by molecular simulations of complex lipid bilayers. PLoS Comp. Biol. *10*, e1003911.

Kunz, J., Wilson, M.P., Kisseleva, M., Hurley, J.H., Majerus, P.W., and Anderson, R.A. (2000). The activation loop of phosphatidylinositol phosphate kinases determines signaling specificity. Mol. Cell *5*, 1–11.

Kutateladze, T.G. (2010). Translation of the phosphoinositide code by PI effectors. Nat. Chem. Biol. *6*, 507–513.

Lemmon, M.A. (2008). Membrane recognition by phospholipid-binding domains. Nat. Rev. Mol. Cell Biol. *9*, 99–111.

Liu, A.Z., Sui, D.X., Wu, D.Q., and Hu, J. (2016). The activation loop of PIP5K functions as a membrane sensor essential for lipid substrate processing. Sci. Adv. *2*, e1600925.

Marrink, S.J., Risselada, J., Yefimov, S., Tieleman, D.P., and de Vries, A.H. (2007). The MARTINI force field: coarse grained model for biomolecular simulations. J. Phys. Chem. B *111*, 7812–7824.

McLaughlin, S., Wang, J., Gambhir, A., and Murray, D. (2002). PIP_2 and proteins: interactions, organization, and information flow. Annu. Rev. Biophys. Biomol. Struct. *31*, 151–175.

Michailidis, I.E., Rusinova, R., Georgakopoulos, A., Chen, Y., Iyengar, R., Robakis, N.K., Logothetis, D.E., and Baki, L. (2011). Phosphatidylinositol-4,5-bisphosphate regulates epidermal growth factor receptor activation. Pflugers Arch. *461*, 387–397.

Monticelli, L., Kandasamy, S.K., Periole, X., Larson, R.G., Tieleman, D.P., and Marrink, S.J. (2008). The MARTINI coarse grained force field: extension to proteins. J. Chem. Theory Comput. *4*, 819–834.

Muftuoglu, Y., Xue, Y., Gao, X., Wu, D.Q., and Ha, Y. (2016). Mechanism of substrate specificity of phosphatidylinositol phosphate kinases. Proc. Natl. Acad. Sci. U S A *113*, 8711–8716.

Naughton, F., Kalli, A.C., and Sansom, M.S.P. (2018). Modes of interaction of pleckstrin homology domains with membranes: toward a computational biochemistry of membrane recognition. J. Mol. Biol. *430*, 372–388.

Naughton, F.B., Kalli, A.C., and Sansom, M.S.P. (2016). Association of peripheral membrane proteins with membranes: free energy of binding of GRP1 PH domain with PIP-containing model bilayers. J. Phys. Chem. Lett. *7*, 1219–1224.

Oostenbrink, C., Villa, A., Mark, A.E., and Van Gunsteren, W.F. (2004). A biomolecular force field based on the free enthalpy of hydration and solvation: the GROMOS force-field parameter sets 53A5 and 53A6. J. Comp. Chem. *25*, 1656–1676.

Parrinello, M., and Rahman, A. (1981). Polymorphic transitions in single-crystals - a new molecular-dynamics method. J. Appl. Phys. *52*, 7182–7190.

Rao, V.D., Misra, S., Boronenkov, I.V., Anderson, R.A., and Hurley, J.H. (1998). Structure of type II beta phosphatidylinositol phosphate kinase: a protein kinase fold flattened for interfacial phosphorylation. Cell *94*, 829–839.

Schmidt, M.R., Stansfeld, P.J., Tucker, S.J., and Sansom, M.S.P. (2013). Simulation-based prediction of phosphatidylinositol 4,5-bisphosphate binding to an ion channel. Biochem *52*, 279–281.

Scott, W.R.P., Hunenberger, P.H., Tironi, I.G., Mark, A.E., Billeter, S.R., Fennen, J., Torda, A.E., Huber, T., Kruger, P., and van Gunsteren, W.F. (1999). The GROMOS biomolecular simulation program package. J. Phys. Chem. A *103*, 3596–3607.

Semenas, J., Hedblom, A., Miftakhova, R.R., Sarwar, M., Larsson, R., Shcherbina, L., Johansson, M.E., Harkonen, P., Sterner, O., and Persson, J.L. (2014). The role of PI3K/AKT-related PIP5K1 alpha and the discovery of its selective inhibitor for treatment of advanced prostate cancer. Proc. Natl. Acad. Sci. U S A *111*, E3689–E3698.

Stahelin, R.V., Scott, J.L., and Frick, C.T. (2014). Cellular and molecular interactions of phosphoinositides and peripheral proteins. Chem. Phys. Lipids *182*, 3–18.

Stansfeld, P.J., and Sansom, M.S.P. (2011). From coarse-grained to atomistic: a serial multi-scale approach to membrane protein simulations. J. Chem. Theory Comput. *7*, 1157–1166.

Stark, A.C., Andrews, C.T., and Elcock, A.H. (2013). Toward optimized potential functions for protein-protein interactions in aqueous solutions: osmotic second virial coefficient calculations using the MARTINI coarse-grained force field. J. Chem. Theory Comput. *9*, 4176–4185.

Suhre, K., and Sanejouand, Y.H. (2004). ElNemo: a normal mode web server for protein movement analysis and the generation of templates for molecular replacement. Nucleic Acids Res. *32*, W610–W614.

Wishart, M.J., and Dixon, J.E. (2002). PTEN and myotubularin phosphatases: from 3-phosphoinositide dephosphorylation to disease. Trends Cell Biol. *12*, 579–585.

Zhou, Y., Prakash, P., Liang, H., Cho, K.J., Gorfe, A.A., and Hancock, J.F. (2017). Lipid-sorting specificity encoded in K-Ras membrane anchor regulates signal output. Cell *168*, 239–251.

STAR★METHODS

KEY RESOURCES TABLE

REAGENT or RESOURCE	SOURCE	IDENTIFIER
Software and Algorithms		
Gromacs 4.6	(Hess et al., 2008)	www.gromacs.org
Martini force field 2.1	(de Jong et al., 2013)	www.cgmartini.nl
GROMOS 53a6 force field	(Oostenbrink et al., 2004)	www.gromacs.org/Downloads/User_contributions/Force_fields
VMD 1.9.2	(Humphrey et al., 1996)	www.ks.uiuc.edu/Research/vmd
PIP5K1A	PDB 4TZ7	www.rcsb.org
Modeller	(Fiser and Sali, 2003)	https://salilab.org/modeller/

CONTACT FOR REAGENT AND RESOURCE SHARING

Further information and requests for resources and reagents should be directed to and will be fulfilled by the Lead Contact, Mark Sansom (mark.sansom@bioch.ox.ac.uk).

METHOD DETAILS

CG simulations were performed using the MARTINI 2.1 force field (Marrink et al., 2007; Monticelli et al., 2008) with a 20 fs time step. Particle coordinates were written out every 0.5 ns. Coulombic interactions were shifted to zero between 0 and 1.2 nm. Lennard-Jones interactions were shifted to zero between 0.9 and 1.2 nm. The nearest neighbour list was updated every 10 steps. A Berendsen thermostat (Berendsen et al., 1984) (coupling constant 1ps) and barostat (coupling constant 1ps, compressibility $5x10^{-6}$ bar^{-1}) were used to maintain temperature at 323 K and pressure at 1bar. The LINCS algorithm (Hess et al., 1997) was used to constrain bond lengths.

Atomistic simulations were performed using the GROMOS 53a1 force field (Scott et al., 1999) with a 2 fs time step. Particle coordinates were written out every 20 ps. Lennard-Jones interactions were shifted to zero between 0.9 and 1.2 nm. Long-range electrostatic interactions were treated using the particle-mesh Ewald method (PME) using default parameters pme-order = 4 and ewald-rtol = 10^{-5}, fourierspacing = 0.12. PME was shifted from 0 to 1 nm. (Darden et al., 1993). The nearest neighbour list was updated every 10 steps. A V-rescale thermostat (coupling constant 1ps) and Parrinello-Rahman (Parrinello and Rahman, 1981) barostat (coupling constant 1ps, compressibility $5x10^{-6}$ bar^{-1}) were used to maintain the temperature and pressure. The LINCS algorithm was used to constrain bond lengths.

Modelling

The crystal structure of PIP5K1A was acquired from the Protein Data Bank (PDB: 4TZ7). Both were deposited as dimers so for monomer simulations the PDB file was amended to include only the first monomer. There are four regions missing from the PIP5K1A structure: the initial sequence of residues (residues 1-55), a short turn (residues 154-156) between the β3 and β4, the insert (residues 310-356), and the activation loop (residues 386-401). For this investigation only the short turn and the activation loop were modelled in the structure, using MODELLER (Fiser and Sali, 2003). The same regions were also removed in the dimer structures. In the numbering scheme used in figures, simulation residues 1 to 253 correspond to PDB structure residues 57 to 309. Simulation residues 254 to 324 correspond to PDB structure residues 356 to 426.

Membrane Binding Simulations

Coarse-grained simulations of PIP5K1A monomers were performed using a 7.5 x 7.5 nm^2 area bilayer of the relevant membrane composition. Lipids were exchanged into a preformed 100% POPC bilayer using a local script. The final lipid percentages were 75% POPC, 20% POPS, and 5% PIP. The kinase centre of mass was positioned 8nm away from the bilayer centre of mass in a random orientation. The box (7x7x20 nm^3) was solvated and sodium and chloride ions added to a concentration of ∼0.15 M. The dimer simulations were performed in a similar manner but with a 15 x 15 x 24 nm^3 bilayer. CG simulations were performed using GROMACS 4.6.5 (Hess et al., 2008). Energy minimisation was carried out via steepest descent and the system equilibrated for 5 ns with protein backbone particles restrained. For each model, 25 repeats were made with different initial velocities.

Atomistic simulations were performed by obtaining a snapshot from CG simulation and conversion via CG2AT, a fragment-based approach (Stansfeld and Sansom, 2011). AT-MD simulations carried out using the GROMACS 5.1 software: the system was equilibrated for 1.5 ns with the backbone atoms of the protein restrained, then a production run of 100-1000 ns carried out.

Analysis

VMD (Humphrey et al., 1996) was used for simulation visualisation. Graphs were generated in matplotlib (Hunter, 2007). Analysis was performed as described below:

Membrane Binding

The GROMACS *g_dist* command was used to obtain the distance between the protein centre of mass and the bilayer centre of mass over time.

Protein-Membrane Contacts

The GROMACS *g_mindist* command was first used to obtain a list of distances of every residue to the nearest PIP phosphate group in each simulation frame. The total number of contacts was counted and normalised to the residue with the largest number of contacts, defining a ‘contact’ as a minimum distance of less than 0.7 nm (0.35 nm in the atomistic simulations).

Distance-Orientation Analysis

A reference ‘productively bound’ structure was selected from simulations in which the kinase catalytic site faces the bilayer headgroups. Frames from each simulations trajectory were fitted in the *xy* plane (parallel to the membrane surface). The rotation matrix of these *xy*-aligned frames relative to the reference structure was then obtained using the GROMACS *g_rotmat* tool, and the R_{ZZ} component, giving rotation from the *z* axis (the membrane normal), was recorded. Distances were obtained using the GROMACS *g_dist* tool, taking the *z* component of distance between the protein and lipid centers-of-mass. Corresponding distance and R_{ZZ} values were plotted using a locally written script.

QUANTIFICATION AND STATISTICAL ANALYSIS

Progress of the membrane association simulations was monitored as the distance of the kinase centre of mass from the bilayer centre vs. time (see Figure S1). The distance was averaged over all simulations in the ensemble (for ensemble sizes $N = 5$ or 25, see Table 1). Exponential fits to binding curves were performed using the Scipy *curve_fit* function, as exemplified in Figure S1E.

DATA AND SOFTWARE AVAILABILITY

Coordinates of the final model generated by this study are available as a Supplemental Information (see below).

Structure, Volume *27*

Supplemental Information

Membrane Recognition and Binding by the Phosphatidylinositol Phosphate Kinase PIP5K1A: A Multiscale Simulation Study

Sarah-Beth T.A. Amos, Antreas C. Kalli, Jiye Shi, and Mark S.P. Sansom

Supplementary Figures

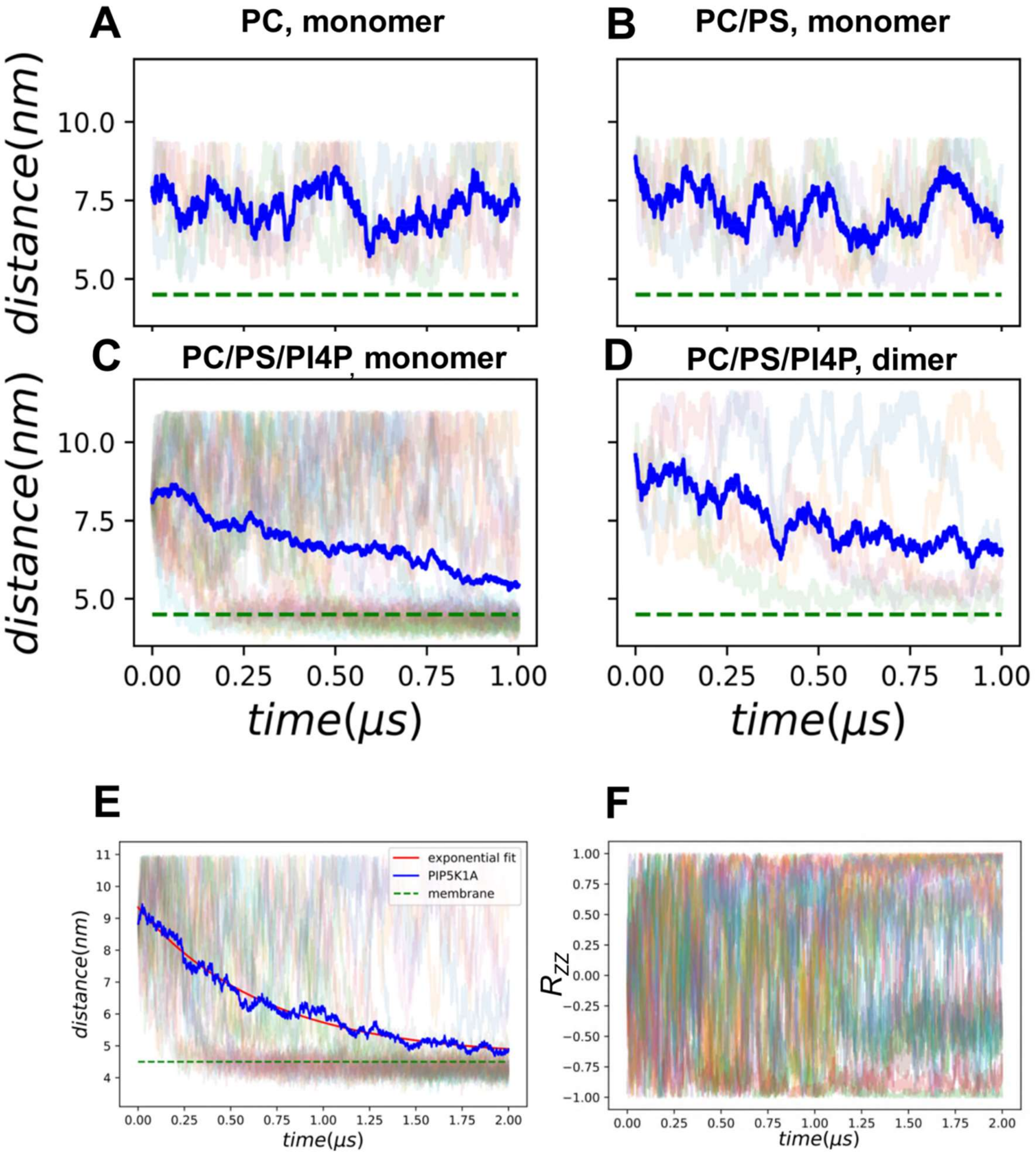

SI Figure S1:

Progress of the membrane association simulations shown as the distance of the kinase centre of mass from the bilayer centre vs. time, averaged over all simulations in the ensemble (see Table 1), for the: **A** PIP5K1A (monomer)/PC; **B** PIP5K1A (monomer)/PC/PS; **C** PIP5K1A (monomer)/PC/PS/PI4P; and **D** PIP5K1A (dimer)/PC/PS/PI4P simulations. The broken horizontal green line indicates the distance at which the kinase would be bound to the bilayer surface. The pale coloured lines represent the distance vs. time data for the individual simulations within each ensemble. **E** PIP5K1A (monomer)/PC/PS/PI4P, 2μs simulations. The average distance of the kinase centre of mass was fitted with a single exponential model (red curve) with a decay time of 0.7 μs. **F** R_{zz} of the individual PIP5K1A monomers over time (shown via a different coloured line for each monomer), demonstrating that the protein tumbles in solution before interacting with the bilayer. (Related to Figure 2.)

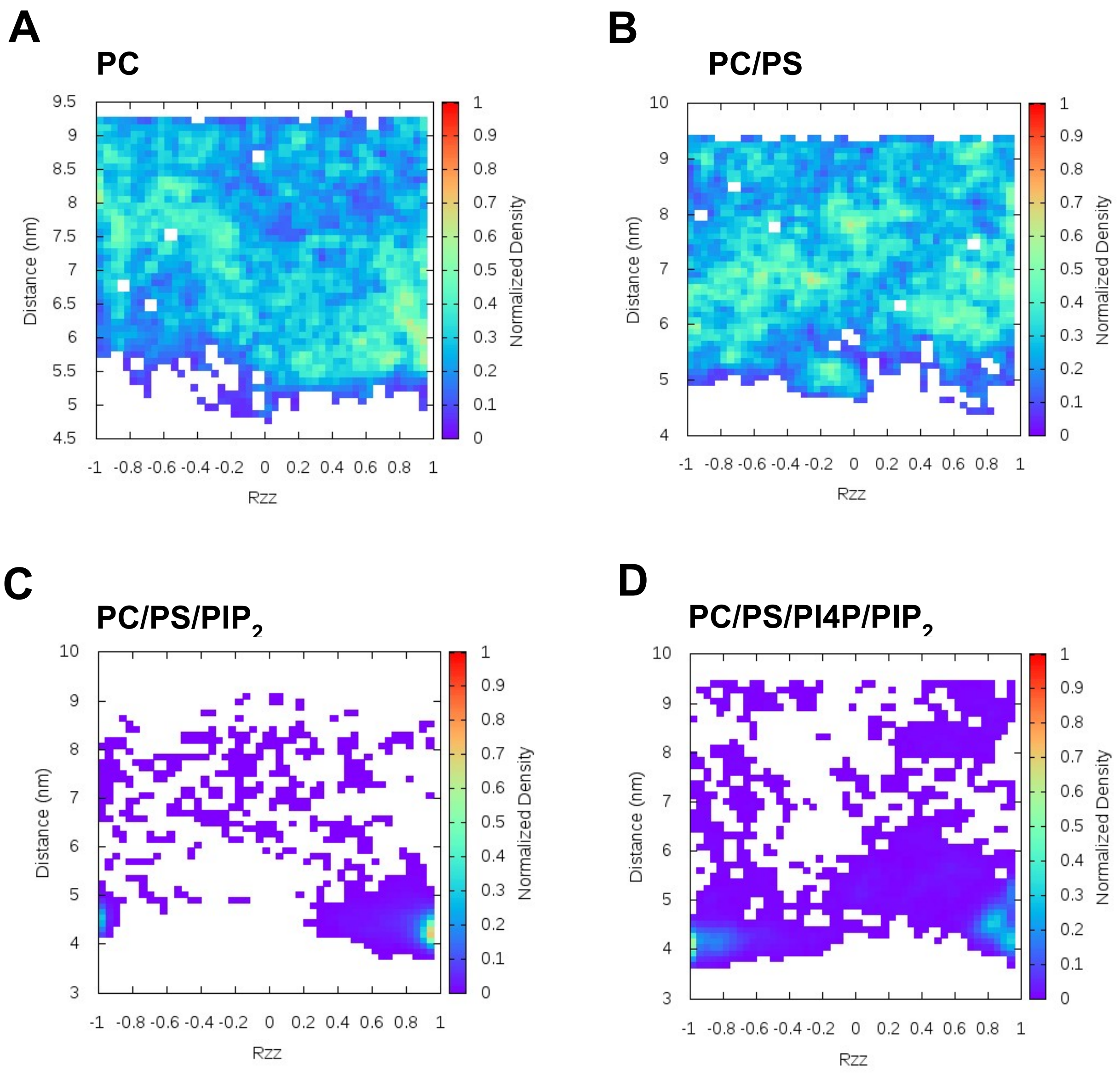

SI Figure S2:

A Orientation density map for PC control; **B** orientation density map for PC/PS; **C** orientation density map for PC/PS/PIP2; **D** orientation density map for PC/PS/PIP2/PI4P mixed. Both control simulations (i.e. **A** and **B**) show a random distribution of orientations, and therefore no specific orientations with respect to the bilayer. (Related to Figure 3.)

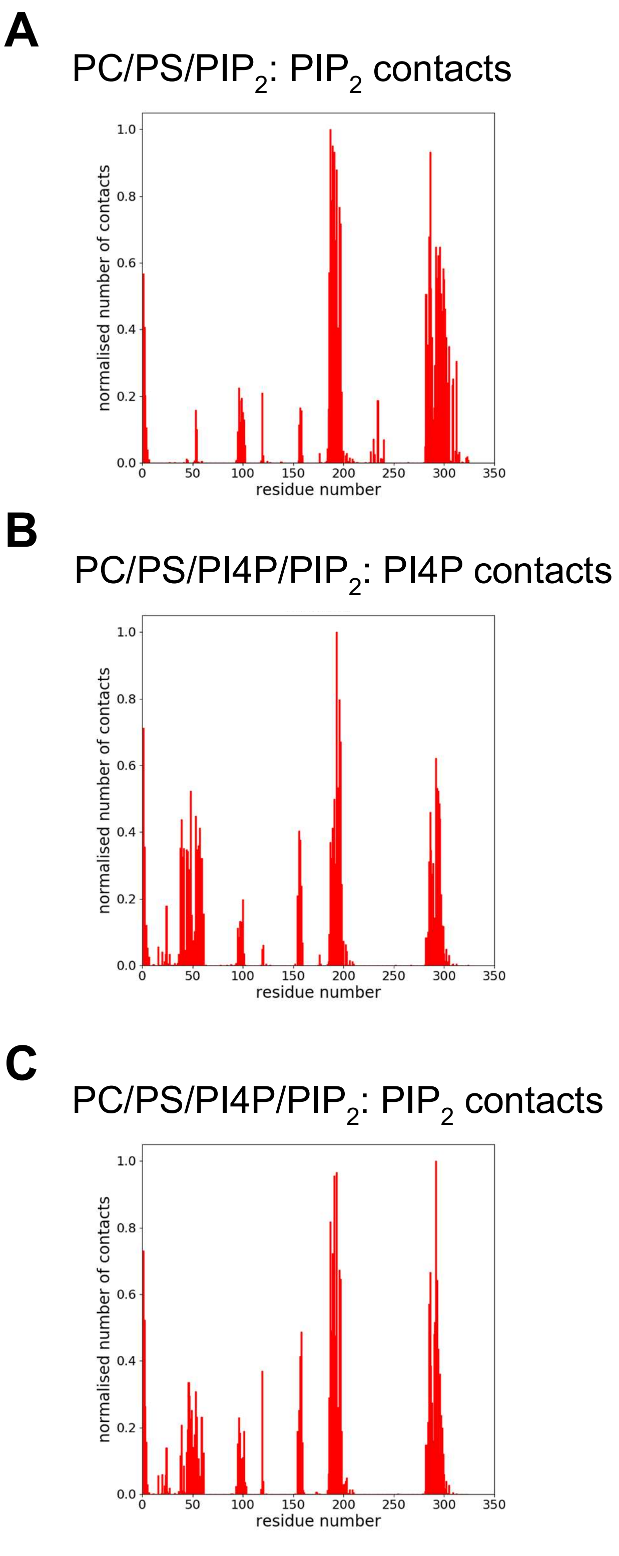

SI Figure S3:

A Normalized number of contacts between PIP5K1A residues and PIP_2 in a PC/PS/PIP_2 membrane; **B** normalized number of contacts between PIP5K1A and PI4P in a PC/PS/PI4P/PIP_2 membrane; **C** normalized number of contacts between PIP5K1A and PIP_2 in a PC/PS/PI4P/PIP_2 membrane. (Related to Figure 3.)

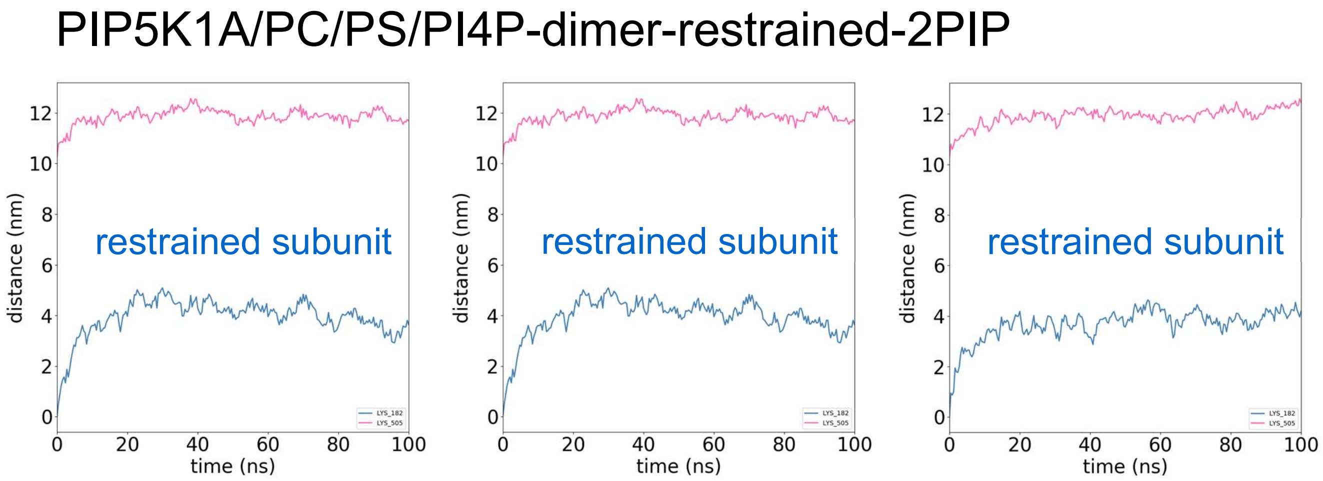

<u>*SI Figure S4:*</u>

Progression of three PIP5K1A/PC/PS/PI4P-dimer-restrained-2PIP (Table 1) simulations performed with the dimer initially positioned flat on the surface of the membrane and restraints placed between the active site lysine residue (PDB residue 328; simulation residue 182) and two PI4P molecules. When K328 residue is restrained to 2 PI4P molecules, the restrained subunit (blue line) remains bound to the membrane and the unrestrained monomer (pink line) remains unbound positioned away from the membrane in the cytoplasm. (Related to Figure 5.)

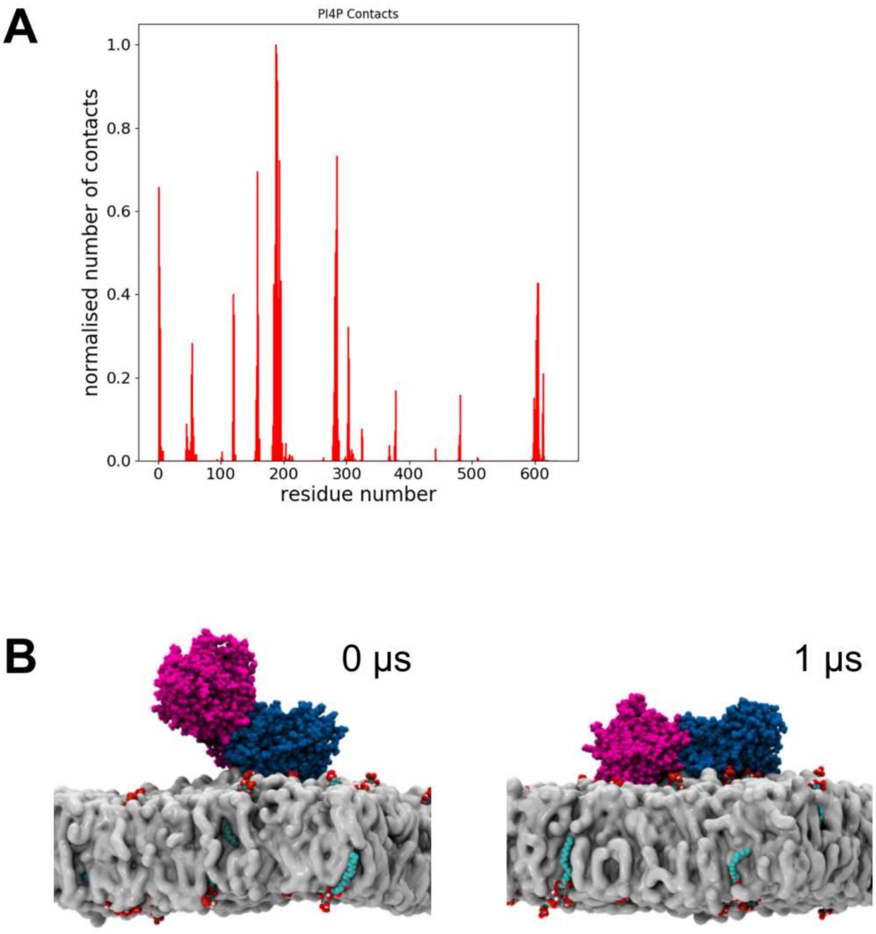

<u>*SI Figure S5:*</u>

A An atomistic PIP5K1A kinase dimer + bilayer system from the AT PIP5K1A/PC/PS/PI4P-dimer simulation (see Table 1) was converted to CG and run for 3 x 1 μs (simulation PIP5K1A/PC/PS/PI4P-dimer-ATconf) to determine whether PI4P could bind to both catalytic sites at the same time. **B** Snapshots of the tilted (0 μs) and flattened orientation of the dimer at the bilayer surface from the AT PIP5K1A/PC/PS/PI4P-dimer simulation. (Related to Figure 6.)

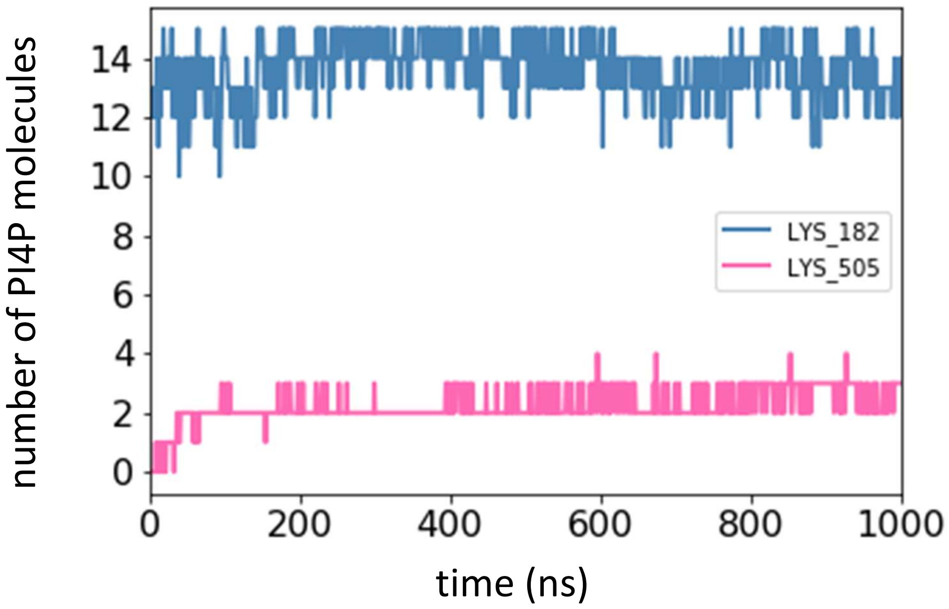

SI Figure S6:

Number of PI4P molecules within 3.5 nm of the active site catalytic residue over the course of the 1 atomistic simulation of the kinase dimer (PIP5K1A/PC/PS/PI4P-dimer). There are 12-14 PI4Ps clustered around the bound monomer, and around 2-4 PIPs near the active site of the other (unbound) monomer. (Related to Figure 6.)
